# Encoding canonical DNA quadruplex structure

**DOI:** 10.1126/sciadv.aat3007

**Published:** 2018-08-31

**Authors:** Scarlett A. Dvorkin, Andreas I. Karsisiotis, Mateus Webba da Silva

**Affiliations:** School of Pharmacy and Pharmaceutical Sciences, Biomedical Sciences Research Institute, Ulster University, Coleraine BT52 1SA, UK.

## Abstract

The main challenge in DNA quadruplex design is to encode a three-dimensional structure into the primary sequence, despite its multiple, repetitive guanine segments. We identify and detail structural elements describing all 14 feasible canonical quadruplex scaffolds and demonstrate their use in control of design. This work outlines a new roadmap for implementation of targeted design of quadruplexes for material, biotechnological, and therapeutic applications.

## Teaser

The remarkable architectural diversity of four-stranded DNA known as G-quadruplexes can be rationally designed.

## INTRODUCTION

In the design of objects composed of nucleic acids, it is crucial to avoid the presence of alternative molecular recognition motifs, since these may result in nontargeted architectures. At the same time, quadruplex DNA is characterized by the presence of repetitive segments of guanines, which have the potential for a great variety of alternative hydrogen bond alignments. The prediction of quadruplex architectural folds encoded in guanine-rich DNA sequence is an important challenge relevant for understanding its significant regulatory roles (*1*), with implications on the evaluation of new therapeutic targets (*2*). The current general consensus is that predicting or controlling quadruplex folding is a mostly intractable problem. For example, biologically significant DNA sequences are often intrinsically polymorphic in vitro and respond to pH, cations, or crowding conditions. Nevertheless, there is substantial interest in resolving this issue due to the potential of these structures as functional materials (*3*, *4*), in templated organization of materials (*5*), as nanowires (*6*), in catalysis (*7*), and as therapeutics (*8*). For example, a quadruplex-based nanomotor relying on a conformational switch between canonical quadruplex and duplex quadruplex resulted in approximately 5-nm displacement (*3*). Potential application of quadruplexes to nanoelectronics has been illustrated by measurements of currents greater than 100 pA in single-molecule quadruplex wires over 100 nm long (*6*). Direct imaging of quadruplex formation and their association with proteins have been enabled by constructing a quadruplex into a DNA origami scaffold (*4*). These examples illustrate the significant utility of the controlled design of quadruplexes. However, the design of quadruplexes is a complex problem also influenced by attributes of the self-assembly environment.

**Fig. 1:**
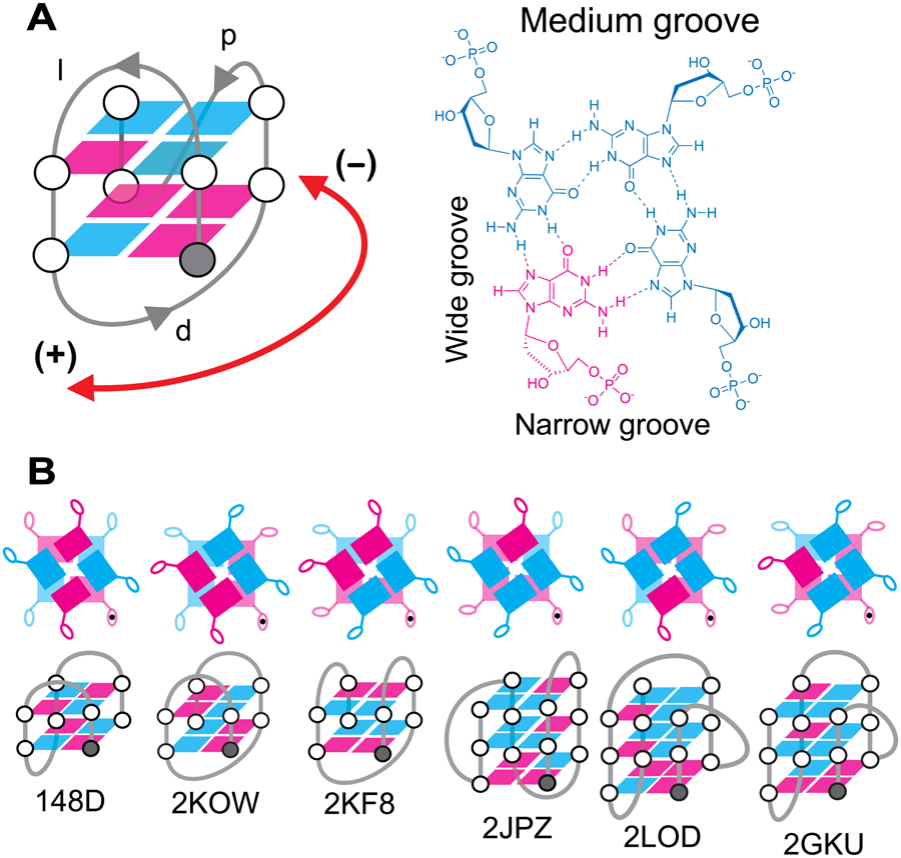
Structural descriptors of canonical quadruplexes. (**A**) The 2(+l_n_d−p) folding topology and hydrogen bond alignments for its top tetrad are shown. Magenta denotes *syn* glycosidic bond angles, and cyan denotes *anti*. The gray circle indicates the 5′ end of the stem. Strand directionalities are indicated by (−) when counter-clockwise and by (+) when clockwise. Propeller loops are indicated by the symbol “p,” diagonal loops by “d,” and lateral loops by “l.” (**B**) Schematic representations of named high-resolution solution structures publicly available in the Protein Data Bank (PDB) (*42*) are shown, with corresponding PDB ID and the respective schematic of the quadruplex topology. The topology of the two-stacked thrombin binding aptamer “TBA” (PDB ID: 148D) is known as a chair-type quadruplex. It can be described as a quadruplex adopting the 2(+l_n_+l_w_+l_n_) topology. The two two-stacked basket-type architectures of human telomeric repeats (2KF8) and Giardia telomeric repeats (2KOW) are denoted 2(−l_w_d+l_n_) and 2(+l_n_d−l_w_), respectively. The three-stacked form-1 and form-2 topologies of human telomeric sequences are described by 3(−p−l_n_−l_w_) for (2GKU) and 3(−l_w_−l_n_−p) for (2JPZ), respectively. Finally, the (3+1) scaffold of 2LOD can be named 3(−pd+l_n_).

A major step in addressing this problem is to develop the ability to define the structural characteristics of quadruplexes. Canonical quadruplexes are composed of a single strand containing at least four tracts of two or more guanines that form a stem. These are linked by three loops, d(G_*n*_L_*x*_G_*n*_L_y_G_*n*_L_*z*_G_*n*_), where *n* is the number of sequential guanines that form the stem, and L is the number (*x*, *y*, or *z*) of residues linking guanines in the stem. In the stem, a guanine from each of the four guanine tracts (G-tracts) engages in hydrogen bonds along its Hoogsteen and Watson-Crick edges to form a tetrad (also known as a quartet). In the stacked tetrads, each of the 2′-deoxyguanosines adopts one of two conformational states that relate base to sugar through the glycosidic bond: the glycosidic torsion angle χ. These two nonoverlapping ranges are described as either *syn* (−90° ≤ χ ≤ +90°) or *anti* (+90° ≤ χ ≤ +180°) conformation. The glycosidic bond angle defines the depth of the grooves along the stem of the molecule. Instead of minor and major grooves found between each strand in double helical DNA, in quadruplexes, the grooves can be described as narrow (n), medium (m), and wide (w) as defined by the glycosidic bond conformation adopted by any of two base-paired 2′-deoxyguanosines of stacked tetrads in the stem (Fig. 1A).

Throughout the publicly available literature, various names have been used to describe canonical quadruplex architecture, including “chair-type,” “basket-type,” “(3+1) scaffold,” “(2+2) scaffold,” “form-1,” and “form-2” telomeric scaffolds. While these terms have had some merit for describing quadruplexes in the past, the structural diversity of quadruplexes can now be classified (*9*, *10*), and therefore systematic nomenclature can be developed. A canonical quadruplex can be denominated by a single descriptor containing the number of guanines in the stem, along with the type and relative direction of loops linking G-tracts of the stem: *n*(L1,L2,L3) (see Fig. 1). Direction of loop progression, L1 to L3, is described in relation to a frame of reference (*9*) beginning with the 5′ end as the lower right corner of stem from the viewer’s perspective. Clockwise progressing loops originating from this frame of reference are denoted with a “+” preceding the loop type, and loops progressing anticlockwise are denoted with a “−” preceding the loop type. Finally, the grooves of the quadruplex stem can be encoded in the description for lateral loops as “w” for wide, “n” for narrow, or “m” for medium grooves, and denoted in subscript after the lateral loop designator. A lateral loop over a narrow groove is thus denoted as l_n_ and over a wide groove as l_w_.

This nomenclature does not directly address the position of the glycosyl bond conformation of the guanosines in the topology. Knowing the precise position of the glycosyl bond conformation of 2′-deoxyguanosines in a quadruplex stem is important for quadruplex design (*11*) and exploitation of their physical properties (*6*, *12*). Eight possible groove type combinations exist, which result from variations of glycosyl bond conformation of guanosines in the stem. Steps of glycosyl bond progressing through a strand of the stem can be either all the same glycosidic bond conformation, that is, *syn*-*syn* or *anti*-*anti* throughout (type 1), or two variations of mixed conformation (*13*, *14*). Individual steps between the two mixed conformations can be either alternating *syn*-*anti*-*syn* (type 3) or two identical conformations, either followed or preceded by the alternate conformation, that is, *syn*-*syn*-*anti* or *anti*-*anti*-*syn* (type 2). The positions of glycosyl bond conformations for type 1, type 2, and type 3 quadruplex stem have been suggested, but some have yet to be demonstrated experimentally (*11*).

Molecular recognition can be encoded in the DNA sequence through the formation of pseudoplanar hydrogen bonding alignments of guanosines. To achieve these alignments, knowledge of the glycosidic bond conformations to be adopted by guanosines throughout the stem of the targeted topology is required. This in turn enables segment length selection of residues in the primary sequence that will form loops in the final architecture. This approach has been previously applied in the design of a quadruplex architecture containing all three loop types (*11*). However, to render the approach more generally applicable, we addressed the remaining questions herein. Specifically, the topologies for all feasible canonical quadruplexes are described, and this knowledge is applied to the design of canonical quadruplex architectures using modified bases. We demonstrate that in the design of quadruplexes, the combination of lengths of loops is dependent not only on the arrangement of the groove widths but also on the number of stacking tetrads of the quadruplex stem.

## RESULTS

### Propeller loops bridge parallel-stranded *syn*G-*syn*G-*anti*G grooves

**Fig. 2:**
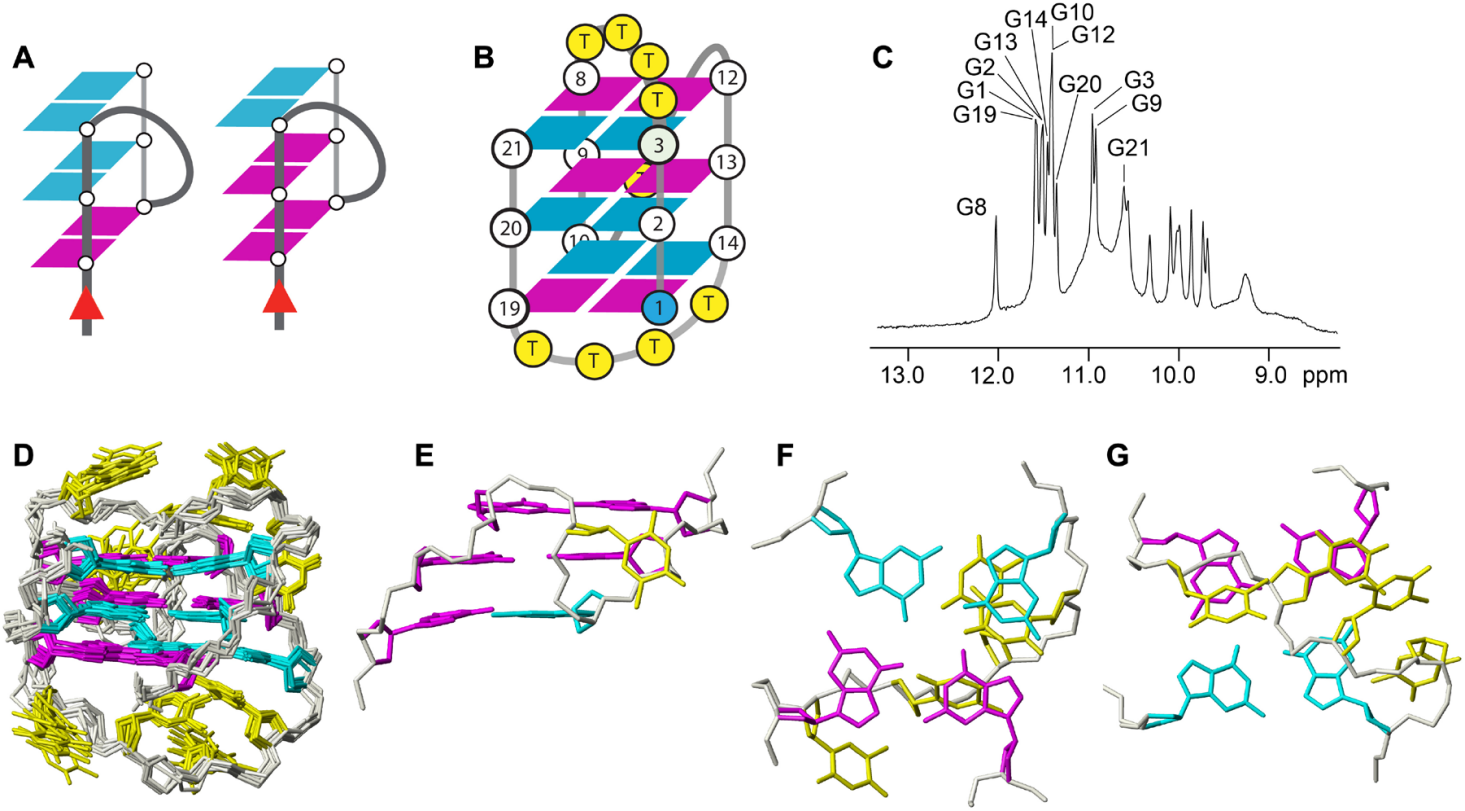
Experimental verification of propeller loops bridging parallel stranded *syn*G-*syn*G-*anti*G grooves. (**A**) Schematic representations of grooves composed of parallel-stranded *syn*G-*anti*G-*anti*G (left) and *syn*G-*syn*G-*anti*G (right) segments. The 2′-deoxyguanosines are shown as *syn* (magenta) and *anti* (cyan) conformations. (B) The design of the 3(d+pd) topology using the DNA sequence d(G_3_T_4_G_3_TG_3_T_4_G_3_), where a single thymine was used to form a propeller loop and four-thymine segments to form diagonal loops. (**C**) Expansion of the proton NMR spectrum at 5°C illustrates the 12 assigned imino protons, indicating formation of a three-stacked quadruplex fold in 16 mM NaCl and 4 mM NaH_2_PO_4_/Na_2_HPO_4_ (pH 6.8). (**D**) Family of 10 superpositioned refined structures of the quadruplex formed by this sequence in solution. (**E**) View into the medium grove populated by the single thymine of the propeller clockwise loop. (**F**) Bird’s eye view of the disposition of the second diagonal loop capping the stem over the (G1:G14:G10:G19) tetrad. (**G**) Bird’s eye view of the disposition of the first diagonal loop over the (G3:G12:G8:G21) tetrad. ppm, parts per million.

To evaluate the feasibility of propeller loop formation spanning grooves composed of parallel-stranded *syn*G-*syn*G-*anti*G, we designed the 3(d+pd) topology and determined its solution structure (Fig. 2) using nuclear magnetic resonance (NMR) spectroscopy. For experimental details, see Supplementary Materials. The sequence d(G_3_T_4_G_3_TG_3_T_4_G_3_) in 20 mM sodium adopts the target 3(d+pd) topology (see Fig. 2, C to F). The right-handed type 2 stem is composed of an equal number of *syn*G and *anti*G. The propeller loop spans a groove composed of parallel-stranded *syn*G-*syn*G-*anti*G, demonstrating that this structural motif is feasible in quadruplex structure.

### The 5′ end of type 3 stem adopts *syn* conformation

**Fig. 3:**
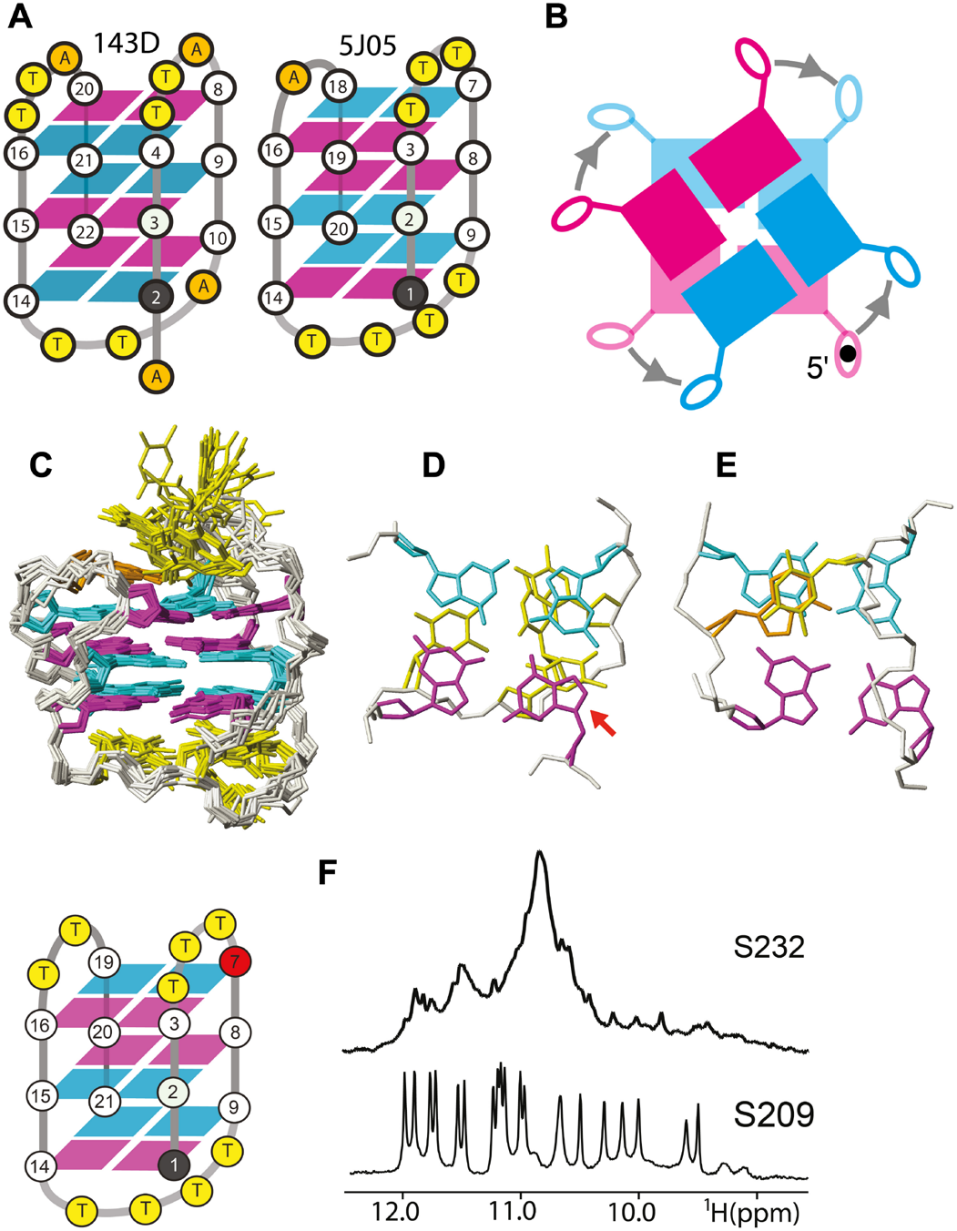
Alternative conformations of glycosyl bonds in a quadruplex stem are possible. (**A**) Schematic representations of the alternative sequence of glycosidic bond angles in the 3(−l_w_d+l_n_) topology for PDB ID 143D (left) and the designed 5J05, as well as corresponding groove-width combinations (**B**) in the stem. (**C**) A bundle of solution structures adopted by the DNA sequence 5J05 in 100 mM sodium solution at pH 6.8. (**D**) The capping of the diagonal onto the (G1:G9:G20:G14) tetrad, with the arrow indicating the position of *syn*G1. (**E**) Detail of the intrastrand stacking of the interdigitated adenine stacking onto *anti*G18 of the stem and T6. (**F**) Design of the 3(−l_w_d+l_n_) topology by replacing the nucleoside dG7 of the DNA sequence S232 by an rG in S209, as shown in red in the schematics for the topology. In the expansion of the proton NMR spectrum in 0.1 M sodium solutions at 5°C shown, the imino protons in the spectrum of S232 appear predominantly as “hump” at approximately 10.8 ppm, indicating that the sequence is mostly unfolded. Nineteen of 21 possible imino protons in the DNA sequence appear in the spectrum of S209, indicating formation of a three-stacked quadruplex fold. The topology has been structurally characterized (Supplementary Materials).

To evaluate whether a three-stacked type 3 quadruplex folds with a *syn*G in the 5′ end of the stem, we designed a DNA sequence targeting the 3(−l_w_d+l_n_) topology (Fig. 3A) and determined its solution structure using NMR spectroscopy. The DNA sequence d(G_3_T_3_G_3_T_4_G_3_TG_3_) (5J05) folds into the 3(−l_w_d+l_n_) topology in 80 mM NaCl and 20 mM NaH_2_PO_4_/Na_2_HPO_4_ (pH 6.8) (Fig. 3, C to E). Its right-handed type 2 stem includes a *syn*G in its 5′ end. To evaluate whether formation of this motif is influenced by a base attached to the 5′ end of the stem, the DNA sequence d(TG_3_T_3_G_3_T_4_G_3_TG_3_) (S093) was also found to adopt the same topology with a *syn*G in the 5′ end of the stem.

### Encoding topology with conformationally locked 2′-deoxyguanosine derivatives

To evaluate whether the formation of the 3(−l_w_d+l_n_) topology in d(G_3_T_3_G_3_T_4_G_3_T_2_G_3_) (S232) can be induced, we replaced the dG7 in this sequence with an rG d(G_3_T_3_**rG**G_2_T_4_G_3_T_2_G_3_) (S209). The sequence S209 folds into a single species in 100 mM sodium solution at pH 6.8 (Fig. 3E) and adopts the desired 3(−l_w_d+l_n_) topology.

### Loop length and number of stacking tetrads are interdependent in design

To evaluate the hypothesized interdependency of loop length combinations and number of stacking tetrads in the programming of quadruplex topologies, we designed two-, three-, and four-stacked quadruplexes of the (−l_w_d+l_n_) topology with lateral loop variations as shown in Table 1 and structurally characterized them using solution NMR spectroscopy (see Supplementary Materials). Sodium solutions were selected since they favor the formation of type 3 stem (*15*).

**Table 1:** Oligonucleotide sequences designed to study folding of the (−l_w_d+l_n_) topology. Guanosines in *syn* conformation appear in bold when determined. PRE, pre-stem residues; POS, post-stem residues; Ms, multiple species; Str, high-resolution structure determined; Top, characterization of (−l_w_d+l_n_) topology by solution NMR methods.

**Name**	**PRE**	**G1**	**L1**	**G2**	**L2**	**G3**	**L3**	**G4**	**POS**	**Status**
S036	**—**	**G**G**G**G	TT	**G**G**G**G	TTTT	**G**G**G**G	T	**G**G**G**G	**—**	Top
S141	**—**	GGGG	TT	GGGG	TTTT	GGGG	TT	GGGG	**—**	Ms
2M6W	**—**	**G**G**G**G	TT	**G**G**G**G	TTTT	**G**G**G**G	AA	**G**G**G**G	**—**	Str
S069	**—**	**G**G**G**G	TT	**G**G**G**G	TTTT	**G**G**G**G	TTT	**G**G**G**G	**—**	Top
S067	**—**	**G**G**G**G	AA	**G**G**G**G	TTTT	**G**G**G**G	TTT	**G**G**G**G	**—**	Top
S064	**—**	GGGG	TTT	GGGG	TTTT	GGGG	A	GGGG	**—**	Ms
5J6U	**—**	**G**G**G**G	TTT	**G**G**G**G	TTTT	**G**G**G**G	AA	**G**G**G**G	**—**	Str
S080	**—**	**G**G**G**G	TTT	**G**G**G**G	TTTT	**G**G**G**G	TTT	**G**G**G**G	**—**	Top
S066	**—**	GGGG	TTTT	GGGG	TTTT	GGGG	AA	GGGG	**—**	Ms
201D	**—**	**G**G**G**G	TTTT	**G**G**G**G	TTTT	**G**G**G**G	TTTT	**G**G**G**G	**—**	(*43*)
230D	**—**	**G**G**G**G	TUTU	**G**G**G**G	TTTT	**G**G**G**G	UUTT	**G**G**G**I	**—**	(*23*)
S025	**—**	GGG	TT	GGG	TTTT	GGG	A	GGG	**—**	Ms
S087	**—**	GGG	TT	GGG	TTTT	GGG	AA	GGG	**—**	Ms
S089	**—**	**G**G**G**	TT	G**G**G	TTTT	**G**G**G**	TTT	G**G**G	**—**	Top
S088	**—**	**G**G**G**	AA	G**G**G	TTTT	**G**G**G**	TTT	G**G**G	**—**	Top
5J05	**—**	**G**G**G**	TTT	G**G**G	TTTT	**G**G**G**	A	G**G**G	**—**	Str
S029	**—**	GGG	TTT	GGG	TTTT	GGG	T	GGG	**—**	Ms
S093	T	**G**G**G**	TTT	G**G**G	TTTT	**G**G**G**	A	G**G**G	**—**	Top
S090	**—**	**G**G**G**	TTT	G**G**G	TTTT	**G**G**G**	AA	G**G**G	**—**	Top
S232	**—**	GGG	TTT	GGG	TTTT	GGG	TT	GGG	**—**	Ms
S209	**—**	**G**G**G**	TTT	(rG)**G**G	TTTT	**G**G**G**	TT	G**G**G	**—**	Top
S210	**—**	GGG	TTT	GGG	TTTT	(rG)GG	TT	GGG	**—**	Ms
S231	**—**	**G**G**G**	TTT	G**G**G	TTTT	**G**G**G**	TTT	G**G**G	**—**	Top
S174	**—**	GGG	TTTT	GGG	TTTT	GGG	TT	GGG	**—**	Ms
S175	**—**	GGG	TTTT	GGG	TTTT	GGG	TTT	GGG	**—**	Ms
S038a	**—**	GGG	TTTT	GGG	TTTT	GGG	TTTT	GGG	**—**	Ms
S166	**—**	GG	TT	GG	TTTT	GG	T	GG	**—**	Ms
S169	**—**	GG	TT	GG	TTTT	GG	TT	GG	**—**	*
S230	A	GG	TT	GG	TTTT	GG	TT	GG	**—**	Ms
S172	**—**	**G**G	TT	**G**G	TTTT	**G**G	TTT	**G**G	**—**	Top
S167	**—**	**G**G	TTT	**G**G	TTTT	**G**G	T	**G**G	**—**	Top
5J4W	**—**	**G**G	TTT	**G**G	TTTT	**G**G	TT	**G**G	**—**	Str
S205	A	GG	TTT	GG	TTTT	GG	TT	GG	**—**	Ms
5J4P	**—**	**G**G	TTT	**G**G	TTTT	**G**G	TTT	**G**G	**—**	Str
S168	**—**	GG	TTTT	GG	TTTT	GG	T	GG	**—**	Ms
S171	**—**	**G**G	TTTT	**G**G	TTTT	**G**G	TT	**G**G	**—**	Top
2M6V	**—**	**G**G	GTTG	**G**G	TTTT	**G**G	GT	**G**G	G	Str
2KF8	**—**	**G**G	GTTA	**G**G	GTTAG	**G**G	TTA	**G**G	GT	(*24*)
2KKA	A	**G**G	GTTA	**G**G	GTTAI	**G**G	TTA	**G**G	GT	(*44*)

*Not (−l_w_d+l_n_). Except for 2KF8 (90 mM K^+^ solution pH 7) and 2KKA (95 mM K^+^ solution pH 7), folding was evaluated in 100 mM Na^+^ solution pH 6.7.

**Fig. 4:**
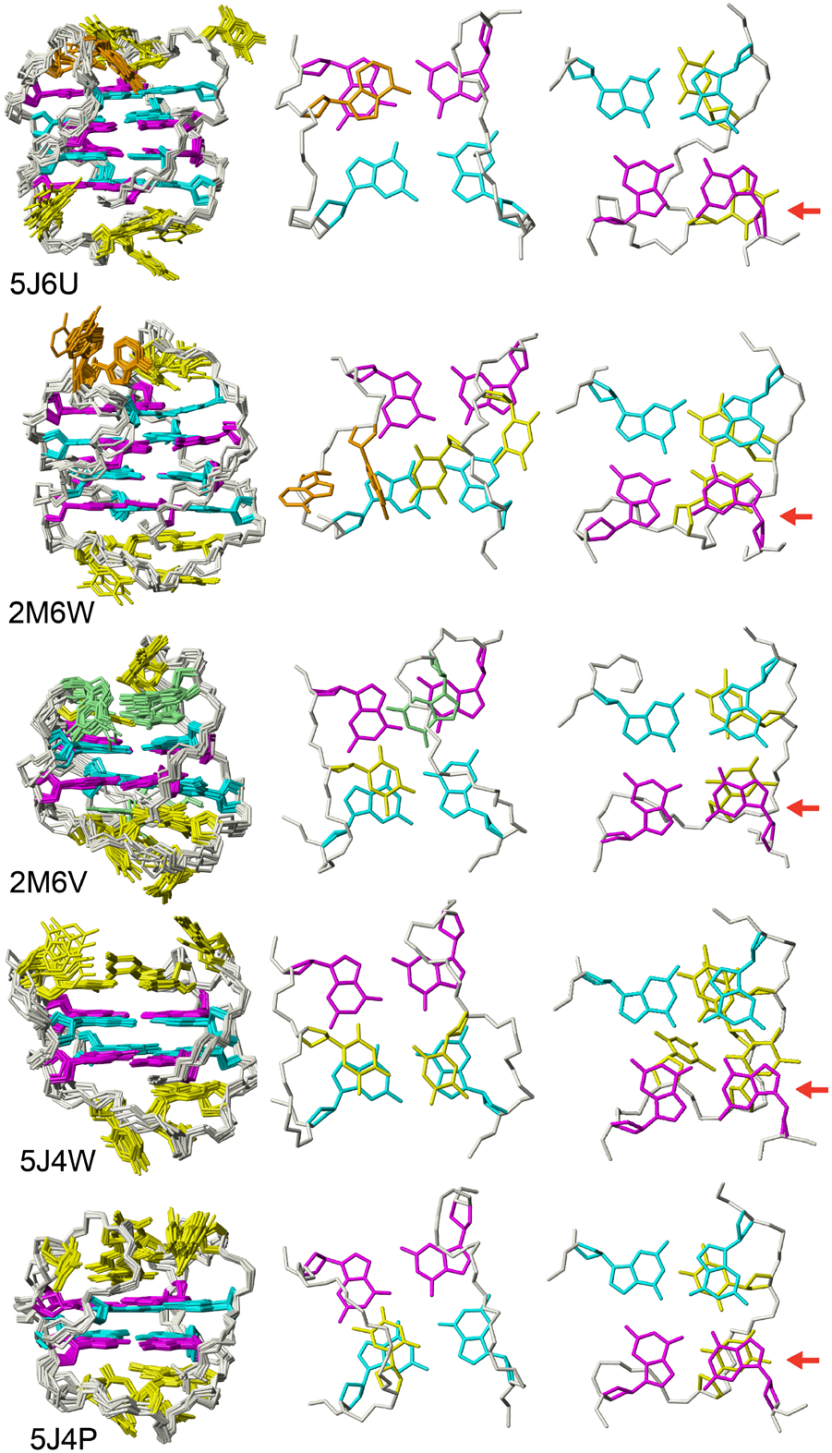
Structural details for high-resolution structures designed to adopt the (−l_w_d+l_n_) topology. The DNA sequences 5J6U and 2M6W adopt 4(−l_w_d+l_n_), and 2M6V, 5J4W, and 5J4P adopt 2(−l_w_d+l_n_) in 100 mM sodium solution at pH 6.8. For each structure, details of loop stacking interactions with the top (left most) and bottom (right) tetrads are depicted. The red arrows indicate proximity of the 5′-*syn*G residue of the stem to the third thymine in the diagonal loop. 2′-Deoxyguanosines of the stem in *syn* (magenta) and *anti* (cyan) conformations, non-stem guanosines (green), and thymines (yellow) are also shown. In all structures, the first thymine of the diagonal loop stacks onto the preceding guanosine of the stem.

The solution structures of DNA sequences d(G_4_T_3_G_4_T_4_G_4_A_2_G_4_) (5J6U),d(G_4_T_2_G_4_T_4_G_4_A_2_G_4_) (2M6W), d(G_3_T_2_G_3_T_4_G_3_TG_3_) (2M6V), d(G_2_T_3_G_2_T_4_G_2_T_2_G_2_) (5J4W), and d(G_2_T_3_G_2_T_4_G_2_T_3_G_2_) (5J4P) were determined, and details are presented in Fig. 4. All sequences form right-handed quadruplexes with a *syn*G at the 5**′** end of the stem and alternation of glycosidic bonds along guanosine segments. The four-thymine diagonal loop adopts a very similar structural environment in all of them, with lateral loop residues stacking onto the stem. The third thymine of the loop stacks onto the first (5′ end) *syn*G of the stem. The close proximity of these two residues was used for initial identification of successful formation of the topology for those sequences that folded successfully in solution and adopt the *n*(−l_w_d+l_n_) topology.

For four-stacked architectures, the data suggest that a difference of more than one residue between the first and third loops prevents folding (Table 1). Combinations of loops d(G_4_T_2_G_4_T_4_G_4_TG_4_) (S036), d(G_4_T_2_G_4_T_4_G_4_A_2_G_4_) (2M6W), d(G_4_T_2_G_4_T_4_G_4_T_3_G_4_) (S069), d(G_4_A_2_G_4_T_4_G_4_T_3_G_4_) (S067), d(G_4_T_3_G_4_T_4_G_4_A_2_G_4_) (5J6U), d(G_4_T_3_G_4_T_4_G_4_T_3_G_4_) (S080), and d(G_4_T_4_G_4_T_4_G_4_T_4_G_4_) (201D), but not d(G_4_T_3_G_4_T_4_G_4_AG_4_) (S064) and d(G_4_T_4_G_4_T_4_G_4_A_2_G_4_) (S066), are able to form 4(−l_w_d+l_n_).

When inducing three-stacked quadruplexes, the probability of forming a propeller loop with combinations of shorter loops is higher (*15*, *16*). For three-stacked architectures, combinations of short loops d(G_3_T_2_G_3_T_4_G_3_AG_3_) (S025) and d(G_3_T_2_G_3_T_4_G_3_A_2_G_3_) (S087) form multiple species in solution. Just as for the 4(−1_w_d+1_n_), only combinations in which the numbers of residues of both loops do not differ by more than a single residue were successful, that is, d(G_3_T_2_G_3_T_4_G_3_T_3_G_3_) (S089), d(G_3_A_2_G_3_T_4_G_3_T_3_G_3_) (S088), d(G_3_T_3_G_3_T_4_G_3_A_2_G_3_) (S090), and d(G_3_T_3_G_3_T_4_G_3_T_3_G_3_) (S231) all adopt the 3(−l_w_d+l_n_) topology. However, in combinations involving four-residue −l_w_ loops, d(G_3_T_4_G_3_T_4_G_3_T_2_G_3_) (S174), d(G_3_T_4_G_3_T_4_G_3_T_3_G_3_) (S175), and d(G_3_T_4_G_3_T_4_G_3_T_4_G_3_) (S038a), multiple species are observed.

Sequences that form 3(−l_w_d+l_n_) exist in which the numbers of residues of both loops differ by more than a single residue, including d(G_3_T_3_G_3_T_4_G_3_AG_3_) 5J05 and its analog d(TG_3_T_3_G_3_T_4_G_3_AG_3_) S093. The analog to 5J05 with a thymine instead of an adenine in the +l_n_ loop, d(G_3_T_3_G_3_T_4_G_3_TG_3_) S029, forms multiple architectures in solution. Inspection of the 5J05 solution structure determined in this study suggests that the stabilization of this architecture may be due to stacking of the single adenine of the +l_n_ loop onto its subsequent *anti*G in the stem.

Short loops such as in d(G_2_T_2_G_2_T_4_G_2_TG_2_) S166 and d(AG_2_T_2_G_2_T_4_G_2_T_2_G_2_) S230 failed to form 2(−l_w_d+l_n_), resulting in multiple species. However, with two residues per lateral loop, d(G_2_T_2_G_2_T_4_G_2_T_2_G_2_) S169 resulted in a single species but could not be characterized as 2(−l_w_d+l_n_). Just as for the 4(−l_w_d+l_n_) and 3(−l_w_d+l_n_), combinations in which the numbers of residues of both loops do not differ by more than a single residue, d(G_2_T_2_G_2_T_4_G_2_T_3_G_2_) S172, d(G_2_T_3_G_2_T_4_G_2_T_2_G_2_) 5J4W, and d(G_2_T_3_G_2_T_4_G_2_T_3_G_2_) 5J4P, adopted the desired 2(−l_w_d+l_n_) topology. Like the 3(−l_w_d+l_n_), but in contrast to 4(−l_w_d+l_n_) sequences, d(G_2_T_3_G_2_T_4_G_2_TG_2_) S167 and d(G_2_T_4_G_2_T_4_G_2_TG_2_) S168 form 2(−l_w_d+l_n_) despite having a two-residue difference between the opposing lateral loops.

An attempt to fold loop combination 2M6V into 3(−l_w_d+l_n_) proved unsuccessful. Instead, it adopted 2(−l_w_d+l_n_), with a four-residue −l_w_ and a two-residue +l_n_ (see Fig. 4). In place of the designed two-residue −l_w_, the 2(−l_w_d+l_n_) has a four-residue loop that incorporates guanosines of the first and second G-rich segments. Thus, the energetically favored architecture still contains the expected diagonal loop formed by four residues, but it also includes a longer (four-residue) loop for −l_w_. While the guanosine of the first G-rich segment forms a *syn*G:T mismatch, the guanosine of the second G-rich segment stacks perfectly onto its preceding *anti*G of the stem. This stabilization motif is also observed for two-residue (2M6W), three-residue (5J6U and 5J4W), and four-residue (2KF8 and 2KKA in Table 1) −l_w_ loops that proceed from a *syn*G to an *anti*G of the stem. The G6:T mismatch appears to be an essential stabilizing factor, since it is absent in the analogous DNA sequences that do not fold (S025).

The −l_w_ loop in two- and four- but not three-stacked (−l_w_d+l_n_) can be stabilized by four residues. However, 3(−l_w_d+l_n_) can be stabilized by a three-residue −l_w_ loop. This may be due to the fact that while in 2(−l_w_d+l_n_) and 4(−l_w_d+l_n_) the −l_w_ loop progresses from an *anti*G to a *syn*G of the stem, the reverse is true for 3(−l_w_d+l_n_). For a −l_w_ loop, a three-residue *syn*G to *anti*G strand progression may be considered mechanically equivalent to a four-residue *anti*G to *syn*G strand progression.

## DISCUSSION

The challenge in design of quadruplexes is to determine the optimal sequence that reliably encodes for a given three-dimensional (3D) structure. Therefore, knowledge of structural motifs of the desired architecture is fundamental to this process. Twenty-six theoretical quadruplexes have been previously proposed (*9*, *11*, *14*); however, a fragment-based molecular mechanics approach applied to the derivation of three-stacked quadruplexes predicted that only 14 of these are mechanically feasible (*17*). Here, we use known and hypothesized glycosyl bond conformations to design 3D architectures to (i) provide experimental verification of all feasible glycosyl bond conformations in these 14 quadruplex topologies and (ii) demonstrate interdependency between loop-length combination and the number of stacking tetrads of quadruplexes.

**Fig. 5:**
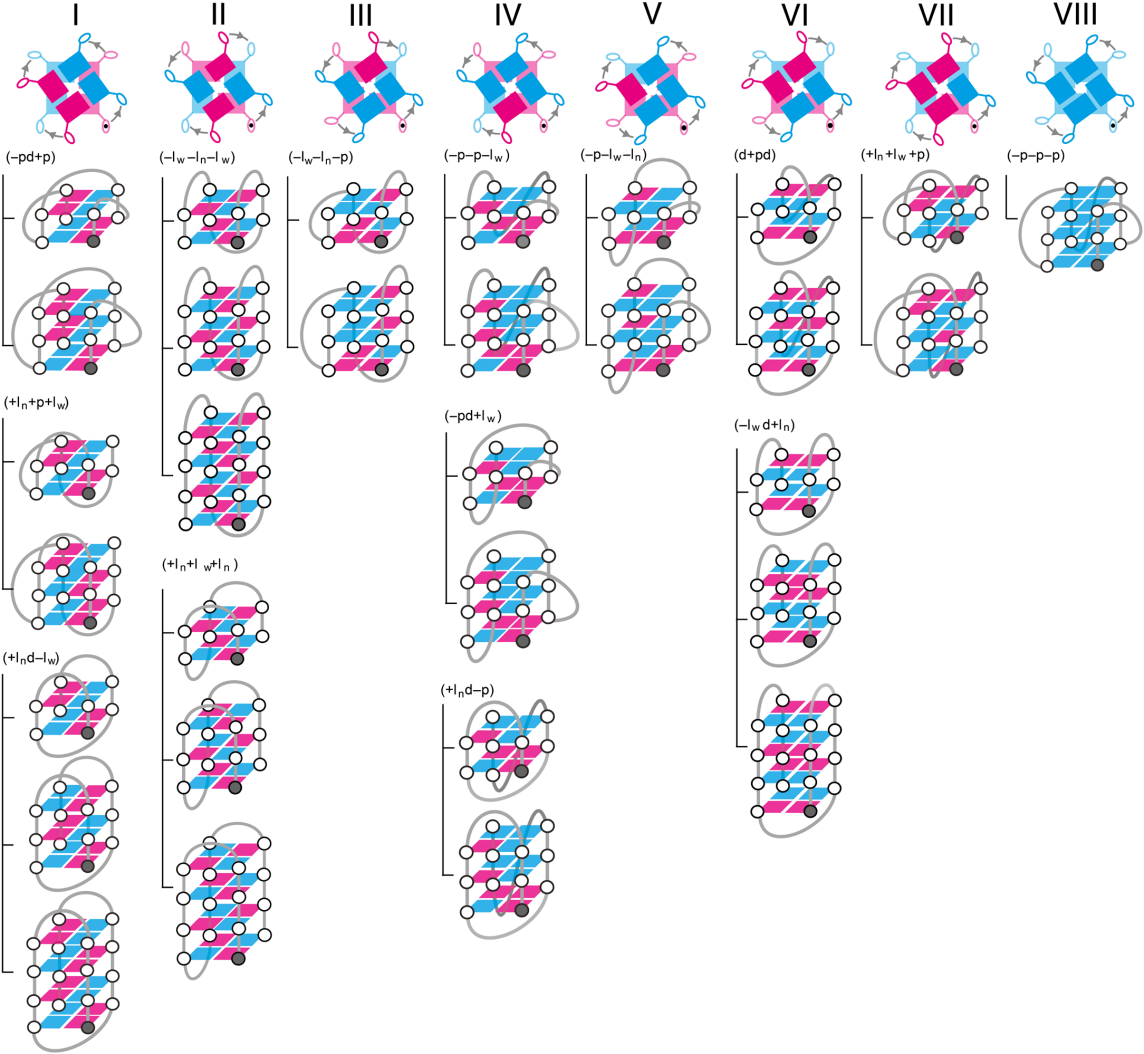
Schematic description of all canonical quadruplex topologies feasible with their constituent groove type combination. (Top) *syn* (magenta) and *anti* (cyan) guanosines combined through hydrogen bond alignments to form tetrads representing the eight possible quadruplex stems. Indicated in gray are the only possible strand progression directionalities for each stem.

In Fig. 5, the schematic representations of glycosidic bond conformations for 14 feasible canonical quadruplex topologies are summarized. Only four of nine type 2 quadruplexes have been experimentally verified: 3(−p−l_w_−l_n_) (*18*), 3(−l_w_−l_n_−p) (*19*), 3(−pd+l_n_) (*20*), and 3(+l_n_+p+l_w_) (*21*). In all four of these topologies, propeller loops bridge grooves of parallel-stranded *syn*G-*anti*G-*anti*G, demonstrating that formation of the previously hypothesized (*11*), but as yet unobserved, topologies 3(+l_n_d−p), 3(−p−p−l_w_), and 3(+l_n_+p+p) is possible. The two remaining type 2 topologies, 3(−pd+p) and 3(d+pd), have a propeller loop bridging a medium groove of parallel-stranded *syn*G-*syn*G-*anti*G. The propeller loop spanning this groove has been confirmed in this study, thus verifying the hypothesized glycosyl bond conformation. Therefore, this structural motif permits formation of the yet to be observed 3(−pd+p). We propose here that type 2 quadruplexes are restricted to two- and three-stacked topologies, since formation of propeller loops for four-stacked canonical type 2 quadruplexes is improbable.

All four type 3 quadruplex topologies shown in Fig. 5 have been experimentally verified. For all two- and four-stacked, the 5′ end of the stem is *syn*G: (+l_n_d−l_w_) (*22*), (−l_w_d+l_n_) (*23*, *24*), (+l_n_+l_w_+l_n_) (*25*, *26*), and (−l_w_−l_n_−l_w_) (*27*). In these architectures, more s*yn*-*anti* steps are present than the less stabilizing (*28*) *anti*-*syn* steps. However, a single three-stacked structure exists with an *anti*G in the 5′ end of the stem: the human telomeric sequence d[AG_3_(T_2_AG_3_)_3_] (*29*), which adopts the topology 3(−l_w_d+l_n_) in 100 mM sodium solution at pH 6.8. Nonetheless, a *syn*G conformation has been suggested to be more stabilizing for a quadruplex stem (*30*). Furthermore, in any three-stacked type 3 quadruplex, the number of *syn*-*anti* steps will be the same as that of *anti*-*syn*, regardless of the conformation adopted by the 5′-stem guanosine. All seven sequences designed to fold 3(−l_w_d+l_n_) topology were observed to adopt a *syn*G at the 5′ end of the quadruplex stem, thus demonstrating it to be the most stable conformation. Other type 3 topologies may also adopt two alternative dispositions of glycosyl bond conformation. The preference for *anti*G at the 5′ end of the stem observed in the human telomeric sequence may outmatch the general preference for *syn*G through the additional stacking of triads or other mismatch alignments onto the type 3 stem.

Individual loop length does not define successful design; it is their combination that does so. For example, although four residues in the first or one residue in the last loop each separately allow folding of (−l_w_d+l_n_), the combination of the two in a single sequence does not. The difference between lateral loops of 2- and 3(−l_w_d+l_n_) can be up to two residues, but only one residue for 4(−l_w_d+l_n_). Also, only 4(−l_w_d+l_n_) is able to fold with lateral loops of one or two residues.

In addition, we determined that the number of stacking tetrads of the targeted topology predicates the optimal loop-length combination. Combinations of two and three, or just three-residue segments for the lateral loops enabled reliable assembly of any *n*(−l_w_d+l_n_) topology. However, greater versatility is permitted for the design of 2(−l_w_d+l_n_) architectures. These can fold with a −l_w_ of four residues, in contrast to 3(−l_w_d+l_n_), and can accommodate a larger difference of residues between lateral loop lengths, in contrast to 4(−l_w_d+l_n_).

Nucleobase substitutions can be used to induce quadruplex folding; however, not all site substitutions will result in well-folded species (*31*), suggesting that strategies for their use require further development. Guanine to 8-bromoguanine substitution has been shown to induce the folding of *syn*G at the substituted site (*21*, *24*, *32*, *33*), and riboguanosine substitution leads to the folding of *anti*G (*34*, *35*). Our unsubstituted sequence d(G_3_T_3_G_3_T_4_G_3_T_2_G_3_) failed to adopt the expected 3(−l_w_d+l_n_) topology (Table 1), but substitution of a single *anti*G of 3(−l_w_d+l_n_) by the conformationally locked rG enabled successful folding of the targeted topology. Although a number of *anti*G positions could have been selected for this substitution, we chose the position at the end of the first loop. This exemplifies how identification of the glycosidic bond conformation of guanosines in the stem enables successful design.

The structural details of canonical quadruplex topologies provided give the explicit information required to design architectures for each topology. This will inform both experimental and theoretical approaches, resulting in improved reliability and reproducibility in quadruplex design. Many principles of design are already established that make it possible to engineer the architectures described. For example, in canonical quadruplex architectures, positioning of loops (as first, second, and third) is fundamental in sequence design, that is, reversing the loop-length sequence in the combination of loops does not result in the same topology (*36*). The nature of residues able to realize the desired loop combinations is equally significant. Here, we used predominantly thymines, and less often adenines, while avoiding cytosines to prevent formation of G:C base pairs. Inclusion of purines in loops may be used to facilitate tetrad stacking and formation of hydrogen bond alignments that should also stack onto tetrads of the quadruplex stem. Successful formation of type 1 quadruplexes is favored in potassium, as well as type 2 and type 3 in sodium solutions (*15*). Potassium can also be used to fold type 2 and type 3 architectures.

Here, we have used four residues to generate diagonal loops. Molecular dynamics (MD) simulation studies (*28*, *37*) and experimental studies (*15*) show that formation of propeller loops is favored by one or two residues. A combination of at least two such loop lengths is likely to be successful for type 1 topology. Loops of two and three residues favor (l_n_) over (l_w_), while four-residue loops favor (l_w_) (*28*). However, lack of loop selectivity for single propeller loops and clockwise lateral loops present unique challenges in design. To mitigate these problems, successful design can be further enabled by oligonucleoside modification strategies as demonstrated in this study. However, more general solutions can be sought from a greater understanding of the prefolding states and folding pathways. This will allow for the development of strategies to control thermodynamic parameters modulating the kinetic routes for self-assembly.

Here, we describe the feasible canonical quadruplex topologies and structural requirements for their design. These scaffolds can be used to inform the design of quadruplex architectures, as well as fine-tune a desired topology for specific applications.

Potential applications in therapeutics and nanotechnology of DNA quadruplexes are varied and diverse. Quadruplex-forming DNA sequences are highly prevalent in mammalian and bacterial genomes where they have established regulatory roles. Small molecules that can stabilize these architectures may be used as therapeutics and sensors (*2*). The structural diversity library based on the set of 14 topologies described here can be interrogated by small-molecule combinatorial libraries for the discovery of leads against the quadruplex topologies represented. Conversely, the quadruplex structural diversity library can also be used to “fish” for protein interaction partners in nuclear extracts to identify quadruplex binding proteins. Quadruplex topologies identified in this manner can then be further fine-tuned for the development of therapeutics, biomarkers, or other diagnostic purposes.

Knowledge of the 14 feasible canonical topologies allows for selection of quadruplex type with structural characteristics appropriate for the loading of desired payloads. These topologies may have potential for use in future drugs for delivery to target locations through stimuli-responsive conformational change involving quadruplex structure and random single-stranded sequence. For example, superparamagnetic nanoparticles surface-functionalized with canonical quadruplexes carrying a payload for intracellular delivery can be thermally activated when subjected to an alternating magnetic field (*38*). Design of quadruplexes can be tailored to create temperature-dependent unfolding (payload delivery) dependent on a range of desired temperatures. The approach can also be used for the construction of devices for targeted delivery to solid tumors.

Quadruplex nanodevices are known to be able to sense cations, small molecules, and proteins [reviewed in (*39*)]. Reversible quadruplex folding can therefore also be controlled through photoregulation or presence of specific cations. For example, in the presence of hemin, quadruplexes are able to catalyze hydrogen peroxide–mediated oxidation of 2,2′-azinobis(3-ethylbenzothiazoline-6-sulfonic acid) to produce a color change which leads to chemiluminescence. Target recognition by the quadruplex is thus observed colorimetrically or through a fluorescence signal. The added ability to tune quadruplex structure in this context will be a powerful tool for a variety of applications, which are expected to include the identification of pathogens and development of diagnostics and biomarkers.

The availability of feasible canonical topologies can also be used to inform the rational design of well-defined quadruplex nanowires (*40*). In principle, systematic design should allow for the modulation of electronic and photoelectronic properties of these materials due to their π-stacking system. This should ultimately enable the fine-tuning of properties that make these architectures good candidates for integrated multicomponent systems.

In summary, programming canonical quadruplex structure based on knowledge of the structural characteristics of the stem has been demonstrated. The complete description of all glycosyl bond angle conformations for canonical quadruplex topologies is presented for the first time. We also demonstrate the feasibility of alternative glycosyl bond conformations within a quadruplex topology. Optimal individual loop lengths have been shown to be dependent on the context of other loop types, as well as loop lengths, within the sequence. Finally, we have shown that the target number of stacking tetrads influences the combination of loop lengths required for successful folding. The improved ability presented here to reliably engineer high-fidelity, topology-specific quadruplex architectures enables the exploitation of this molecule’s unique potential.

## MATERIALS AND METHODS

### Sample preparation

Primary DNA sequences were synthesized, trityl-off, by Eurogentec (Belgium), supplied desalted, and lyophilized. They were subsequently purified by reverse-phase high-performance liquid chromatography in ion-pair mode on a Phenomenex Clarity Oligo-RP column (C18, 5 μm, 10 × 100 mm) using an acetonitrile gradient (5 to 40% over 35 min) and a 100 mM TEAA (triethylamine acetate) buffer (pH 6.8). Typically, the recovered fractions were subsequently subjected to gel filtration using Sephadex G-15 (Sigma-Aldrich) and to, finally, three rounds of dialysis in water/sodium-buffered solutions. After lyophilization, samples were resuspended in concentrations of total sodium ranging from 20 to 100 mM. All solutions were composed of NaCl and Na_2_HPO_4_/NaH_2_PO_4_ buffered to pH 6.8.

### Solution NMR assignments

Proton assignments were performed following well-established procedures and, in some cases, aided by inosine substitutions. Identification of intranucleotide anomeric signals was derived from DQF-COSY (double quantum-filtered correlation spectroscopy) and TOCSY (total correlation spectroscopy) experiments. G-quadruplex sequence-specific assignments were based on sequential nuclear Overhauser effect (NOE) connectivities of the type H8/H6(i)-H1’(i)-H8/H6(i+1) and the corresponding sequential connectivities on H2′/H2″ and H3′ spin systems derived from nuclear Overhauser effect spectroscopy (NOESY) experiments. JR-NOESY experiments allowed for GH1 and GH21/GH22 assignments. Typically, (^1^H-^31^P)HSQC (heteronuclear single quantum correlation) experiments that allow for tracing intranucleotide spin system connectivities involving H3′(i-1)-P(i)-H4′/H5′/H5″(i) were acquired to support or identify residue sequential connectivities. In selected cases, unambiguous assignment of imino H1 from the aromatic H8 guanines in the stem was also performed from natural abundance JR [^1^H-^13^C] HMBC (heteronuclear multiple-bond correlation) experiments. The chemical shift assignments are shown in tables S1 to S7.

### Restraints for structure calculations

Distance restraints were typically derived from NOESY experiments in “100%” ^2^H_2_O at three to five mixing times, and distances estimated from the initial buildup rates of the NOE curves by the two-spin approximation *r*_*ij*_ = *r*_ref_(*R*_*ij*_/*R*_ref_)^1/6^, where *r*_*ij*_ is the distance between protons *i* and *j*, *r*_ref_ is a reference distance, and *R*_*ij*_ and *R*_ref_ are the initial buildup rates. Interproton distances were estimated using the average of the volume integral of the distance between H5-methyl in thymine residues, that is, 2.46 Å. Only two limiting mixing times (60 and 200 ms) were used to derive distance restraints from the exchangeable protons collected with jump-and-return NOESY spectra at 5°C in 90% H_2_O, 10% ^2^H_2_O. Distances were assumed to be 3.0 ± 0.8 Å for strong peaks observed in the 60-ms mixing time spectrum, 4.0 ± 1.2 Å for medium cross-peaks observed in the 200-ms mixing time spectrum, and 5.0 ± 1.8 Å for cross-peaks not observed in a 60-ms mixing time spectrum.

### Structure calculations

Distance-restrained structure determinations were carried out using distance constraints from the NMR data. Calculations were performed using XPLOR-NIH (*41*) using the CHARMM force field and adapted for restrained MD (rMD) for nucleic acids. All calculations were executed in vacuo without explicit counterions. Typically, the distance geometry and simulated annealing refinement protocol started from 3000 different structures randomized over all dihedral angles. A number of structures did usually emerge with the same fold and separated from nonconverged structures by large gaps in components of the potential energy function (dihedral angles, van der Waals, NOE violations, and covalent geometry). These sets were subsequently submitted to rMD calculations, performed using random velocities fitting a Maxwell-Boltzmann distribution. The empirical energy function was developed for nucleic acids and treated all hydrogens explicitly. It consisted of energy terms for hydrogen bonding, nonbonded interactions, bonds, bond angles, torsion angles, and tetrahedral and planar geometry, including van der Waals and electrostatic forces. During these computations, both the glycosidic torsion angle χ and planarity restraints were imposed during computations. The final procedure consisted of a total of 53 ps of rMD, including 7 ps of 14 ps from 300 to 1000 K, a 20-ps scale-up of restraints at high temperature, 14 ps of cooling to 300 K, and 12 ps of equilibration rMD, without planarity restraints. The temperature was controlled by coupling the molecules to a temperature bath with a coupling constant of 0.025 ps. The van der Waals term was approximated using the Lennard-Jones potential energy function, and bond lengths involving hydrogens were fixed with the SHAKE algorithm. NOE, dihedral angle restraints, and chemical shifts were deposited in the Biological Magnetic Resonance Bank (BMRB), and structure coordinates have been deposited in the PDB with identification codes 5J4P, 5J05, 5J4W, 5J6U, 2M6W, 2M6V, and 2MFT, as well as in the BMRB with identification codes 30055, 30045, 30056, 30058, 19159, 19158, and 19571, respectively. Structural restraints are shown in tables S8 to S14.

## Supplementary Material

http://advances.sciencemag.org/cgi/content/full/4/8/eaat3007/DC1

## SUPPLEMENTARY MATERIALS

Supplementary material for this article is available at http://advances.sciencemag.org/cgi/content/full/4/8/eaat3007/DC1 Assessment of folding of DNA sequences Characterization of structure Identification of topology NMR chemical shifts tables Structural statistics tables Fig. S1. Expansions of 1D NMR spectra of imino proton regions for DNA sequences folding into quadruplexes in this study. Fig. S2. NMR structure characterization of 2MFT. Fig. S3. NMR structure characterization of aromatic and anomeric regions of 2MW6. Fig. S4. NMR structure characterization of inosine substitutions for 2MW6. Fig. S5. Intraresidue aromatic-imino assignments for guanines in the stem of 2MW6. Fig. S6. Exchangeable proton assignments for the structure of 2M6W. Fig. S7. Nonexchangeable ^1^H and ^31^P assignments for 5J6U. Fig. S8. Exchangeable proton assignments for 5J6U. Fig. S9. Nonexchangeable ^1^H assignments for 5J05. Fig. S10. Exchangeable proton assignments for 5J05. Fig. S11. Sequence-specific assignments for 5J4W. Fig. S12. Exchangeable proton assignments for 5J4W. Fig. S13. Nonexchangeable ^1^H assignments for 5J4P. Fig. S14. Exchangeable proton assignments for 5J4P. Fig. S15. Nonexchangeable ^1^H and ^31^P assignments for 2M6V. Fig. S16. Exchangeable proton perturbations for the inosine substitutions on 2M6V. Fig. S17. Exchangeable proton assignments for 2M6V. Fig. S18. NMR experiments for characterization of the 4(−l_w_d+l_n_) topology formed by the DNA sequences S069, S067, S036, and S080. Fig. S19. Solution NMR experiments for characterization of the 3(−l_w_d+l_n_) topology formed by the DNA sequences S231, S090, S089, S088, and S093. Fig. S20. Solution NMR experiments for characterization of the 2(−l_w_d+l_n_) topology formed by the DNA sequences S167, S171, and S172. Fig. S21. Use of riboguanosines to induce folding of the 3(−l_w_d+l_n_) topology. Fig. S22. Exchangeable proton assignments for 3(−l_w_d+l_n_) topology formed by S090. Table S1. Proton chemical shifts for the structure of 2MFT. Table S2. Proton and phosphorous chemical shifts for structure of 2M6W. Table S3. Proton and phosphorous chemical shifts for structure of 5J6U. Table S4. Proton chemical shifts for the structure of 5J05. Table S5. Proton chemical shifts for the structure of 5J4W. Table S6. Proton chemical shifts for the structure of 5J4P. Table S7. Proton and phosphorous chemical shifts for the structure of 2M6V. Table S8. NMR restraints and structural statistics for the structures of 2MFT. Table S9. NMR restraints and structural statistics for the structures of 2M6W. Table S10. NMR restraints and structural statistics for the structures of 5J6U. Table S11. NMR restraints and structural statistics for the structures of 5J05. Table S12. NMR restraints and structural statistics for the structures of 5J4W. Table S13. NMR restraints and structural statistics for the structures of 5J4P. Table S14. NMR restraints and structural statistics for the structures of 2M6V.
