# Supplementary material for: Encoding canonical DNA quadruplex structure
Source: Sci Adv. 2018 Aug 31;4(8):eaat3007. doi: 10.1126/sciadv.aat3007 (PMC6118410; doi:10.1126/sciadv.aat3007)
Supplement: http://advances.sciencemag.org/cgi/content/full/4/8/eaat3007/DC1 [file supp_4_8_eaat3007__index.html]

Science Advances | Science Advances

## Supplementary Materials

**This PDF file includes:**

- Assessment of folding of DNA sequences
- Characterization of structure
- Identification of topology
- NMR chemical shifts tables
- Structural statistics tables
- Fig. S1. Expansions of 1D NMR spectra of imino proton regions for DNA sequences folding into quadruplexes in this study.
- Fig. S2. NMR structure characterization of 2MFT.
- Fig. S3. NMR structure characterization of aromatic and anomeric regions of 2MW6.
- Fig. S4. NMR structure characterization of inosine substitutions for 2MW6.
- Fig. S5. Intraresidue aromatic-imino assignments for guanines in the stem of 2MW6.
- Fig. S6. Exchangeable proton assignments for the structure of 2M6W.
- Fig. S7. Nonexchangeable 1H and 31P assignments for 5J6U.
- Fig. S8. Exchangeable proton assignments for 5J6U.
- Fig. S9. Nonexchangeable 1H assignments for 5J05.
- Fig. S10. Exchangeable proton assignments for 5J05.
- Fig. S11. Sequence-specific assignments for 5J4W.
- Fig. S12. Exchangeable proton assignments for 5J4W.
- Fig. S13. Nonexchangeable 1H assignments for 5J4P.
- Fig. S14. Exchangeable proton assignments for 5J4P.
- Fig. S15. Nonexchangeable 1H and 31P assignments for 2M6V.
- Fig. S16. Exchangeable proton perturbations for the inosine substitutions on 2M6V.
- Fig. S17. Exchangeable proton assignments for 2M6V.
- Fig. S18. NMR experiments for characterization of the 4(−lwd+ln) topology formed by the DNA sequences S069, S067, S036, and S080.
- Fig. S19. Solution NMR experiments for characterization of the 3(−lwd+ln) topology formed by the DNA sequences S231, S090, S089, S088, and S093.
- Fig. S20. Solution NMR experiments for characterization of the 2(−lwd+ln) topology formed by the DNA sequences S167, S171, and S172.
- Fig. S21. Use of riboguanosines to induce folding of the 3(−lwd+ln) topology.
- Fig. S22. Exchangeable proton assignments for 3(−lwd+ln) topology formed by S090.
- Table S1. Proton chemical shifts for the structure of 2MFT.
- Table S2. Proton and phosphorous chemical shifts for structure of 2M6W.
- Table S3. Proton and phosphorous chemical shifts for structure of 5J6U.
- Table S4. Proton chemical shifts for the structure of 5J05.
- Table S5. Proton chemical shifts for the structure of 5J4W.
- Table S6. Proton chemical shifts for the structure of 5J4P.
- Table S7. Proton and phosphorous chemical shifts for the structure of 2M6V.
- Table S8. NMR restraints and structural statistics for the structures of 2MFT.
- Table S9. NMR restraints and structural statistics for the structures of 2M6W.
- Table S10. NMR restraints and structural statistics for the structures of 5J6U.
- Table S11. NMR restraints and structural statistics for the structures of 5J05.
- Table S12. NMR restraints and structural statistics for the structures of 5J4W.
- Table S13. NMR restraints and structural statistics for the structures of 5J4P.
- Table S14. NMR restraints and structural statistics for the structures of 2M6V.

Download PDF

**Files in this Data Supplement:**

- Adobe PDF - aat3007\_SM.pdf
