# Supplementary material for: Encoding canonical DNA quadruplex structure
Source: Sci Adv. 2018 Aug 31;4(8):eaat3007. doi: 10.1126/sciadv.aat3007 (PMC6118410; doi:10.1126/sciadv.aat3007)
Supplement: http://advances.sciencemag.org/cgi/content/full/4/8/eaat3007/DC1 [file aat3007_SM.pdf]

Science Advances
AAAS

advances.sciencemag.org/cgi/content/full/4/8/eaat3007/DC1

Supplementary Materials for

Encoding canonical DNA quadruplex structure

Scarlett A. Dvorkin, Andreas I. Karsisiotis, Mateus Webba da Silva*

*Corresponding author. Email: mm.webba-da-silva@ulster.ac.uk

Published 31 August 2018, *Sci. Adv.* **4**, eaat3007 (2018)
DOI: 10.1126/sciadv.aat3007

This PDF file includes:

Assessment of folding of DNA sequences
Characterization of structure
Identification of topology
NMR chemical shifts tables
Structural statistics tables
Fig. S1. Expansions of 1D NMR spectra of imino proton regions for DNA sequences folding into quadruplexes in this study.
Fig. S2. NMR structure characterization of 2MFT.
Fig. S3. NMR structure characterization of aromatic and anomeric regions of 2MW6.
Fig. S4. NMR structure characterization of inosine substitutions for 2MW6.
Fig. S5. Intraresidue aromatic-imino assignments for guanines in the stem of 2MW6.
Fig. S6. Exchangeable proton assignments for the structure of 2M6W.
Fig. S7. Nonexchangeable ^{1}H and ^{31}P assignments for 5J6U.
Fig. S8. Exchangeable proton assignments for 5J6U.
Fig. S9. Nonexchangeable ^{1}H assignments for 5J05.
Fig. S10. Exchangeable proton assignments for 5J05.
Fig. S11. Sequence-specific assignments for 5J4W.
Fig. S12. Exchangeable proton assignments for 5J4W.
Fig. S13. Nonexchangeable ^{1}H assignments for 5J4P.
Fig. S14. Exchangeable proton assignments for 5J4P.
Fig. S15. Nonexchangeable ^{1}H and ^{31}P assignments for 2M6V.
Fig. S16. Exchangeable proton perturbations for the inosine substitutions on 2M6V.
Fig. S17. Exchangeable proton assignments for 2M6V.
Fig. S18. NMR experiments for characterization of the $4(-l_wd+l_n)$ topology formed by the DNA sequences S069, S067, S036, and S080.
Fig. S19. Solution NMR experiments for characterization of the $3(-l_wd+l_n)$ topology formed by the DNA sequences S231, S090, S089, S088, and S093.
Fig. S20. Solution NMR experiments for characterization of the $2(-l_wd+l_n)$ topology formed by the DNA sequences S167, S171, and S172.

Fig. S21. Use of riboguanosines to induce folding of the $3(-l_{w}d+l_{n})$ topology.
Fig. S22. Exchangeable proton assignments for $3(-l_{w}d+l_{n})$ topology formed by S090.
Table S1. Proton chemical shifts for the structure of 2MFT.
Table S2. Proton and phosphorous chemical shifts for structure of 2M6W.
Table S3. Proton and phosphorous chemical shifts for structure of 5J6U.
Table S4. Proton chemical shifts for the structure of 5J05.
Table S5. Proton chemical shifts for the structure of 5J4W.
Table S6. Proton chemical shifts for the structure of 5J4P.
Table S7. Proton and phosphorous chemical shifts for the structure of 2M6V.
Table S8. NMR restraints and structural statistics for the structures of 2MFT.
Table S9. NMR restraints and structural statistics for the structures of 2M6W.
Table S10. NMR restraints and structural statistics for the structures of 5J6U.
Table S11. NMR restraints and structural statistics for the structures of 5J05.
Table S12. NMR restraints and structural statistics for the structures of 5J4W.
Table S13. NMR restraints and structural statistics for the structures of 5J4P.
Table S14. NMR restraints and structural statistics for the structures of 2M6V.

Assessment of folding of DNA sequences

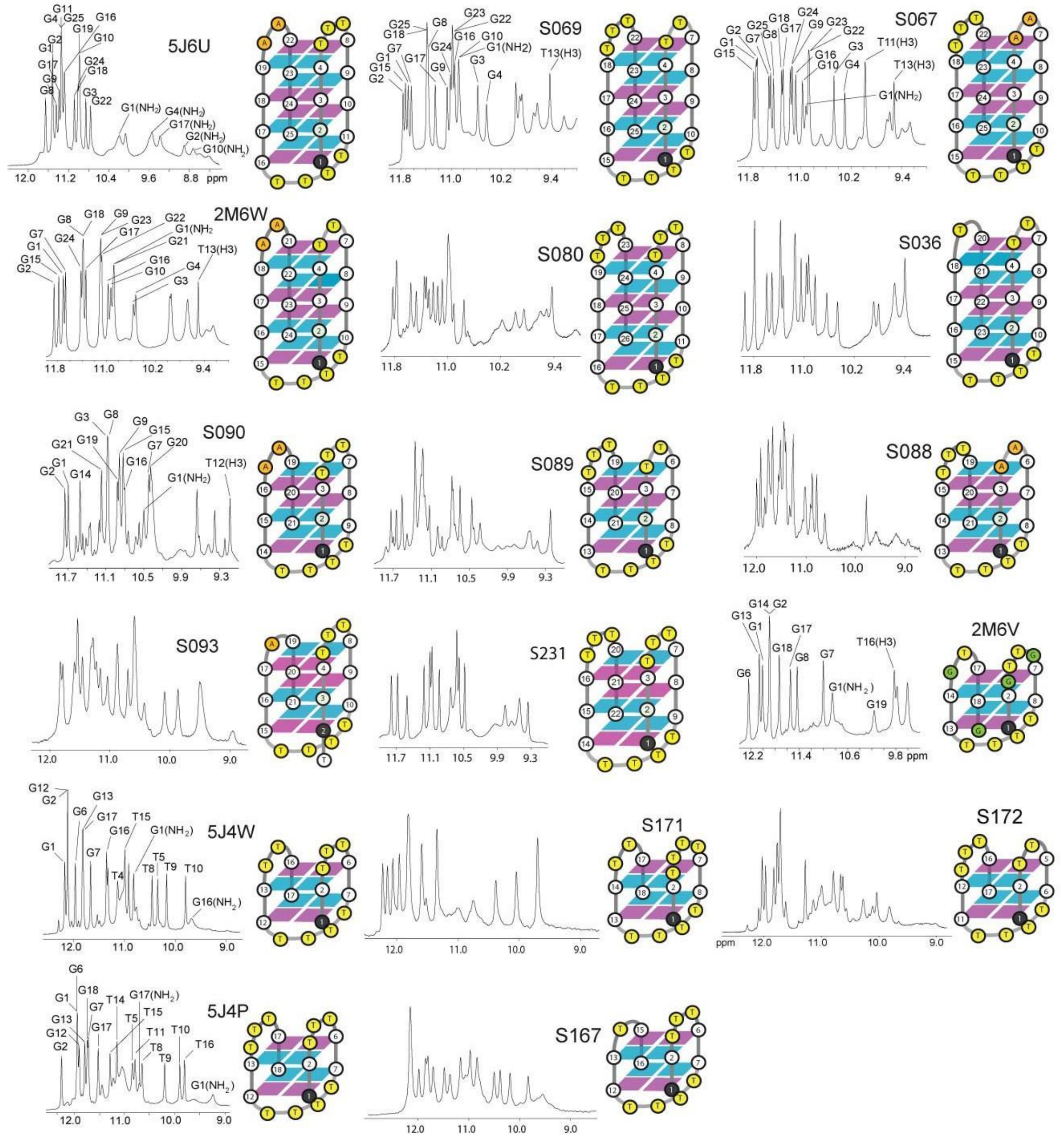

Fig. S1. Expansions of 1D NMR spectra of imino proton regions for DNA sequences folding into quadruplexes in this study. Expansions of 1D NMR spectra of imino proton regions for DNA sequences at ~2 mM oligonucleotide concentrations in 80 mM NaCl, 20 mM NaH_2PO_4/Na_2HPO_4, pH 6.8 solutions at at 5°C. Shown are also schematic representations of the (-l_wd+l_n) topology they adopt with 2’-deoxyguanosines of the stem in *syn* (magenta) and *anti* (cyan) conformations, green for non-stem guanosines, and orange for adenines, and yellow for thymines.

Characterization of structure

Structural assignments for 2MFT in sodium

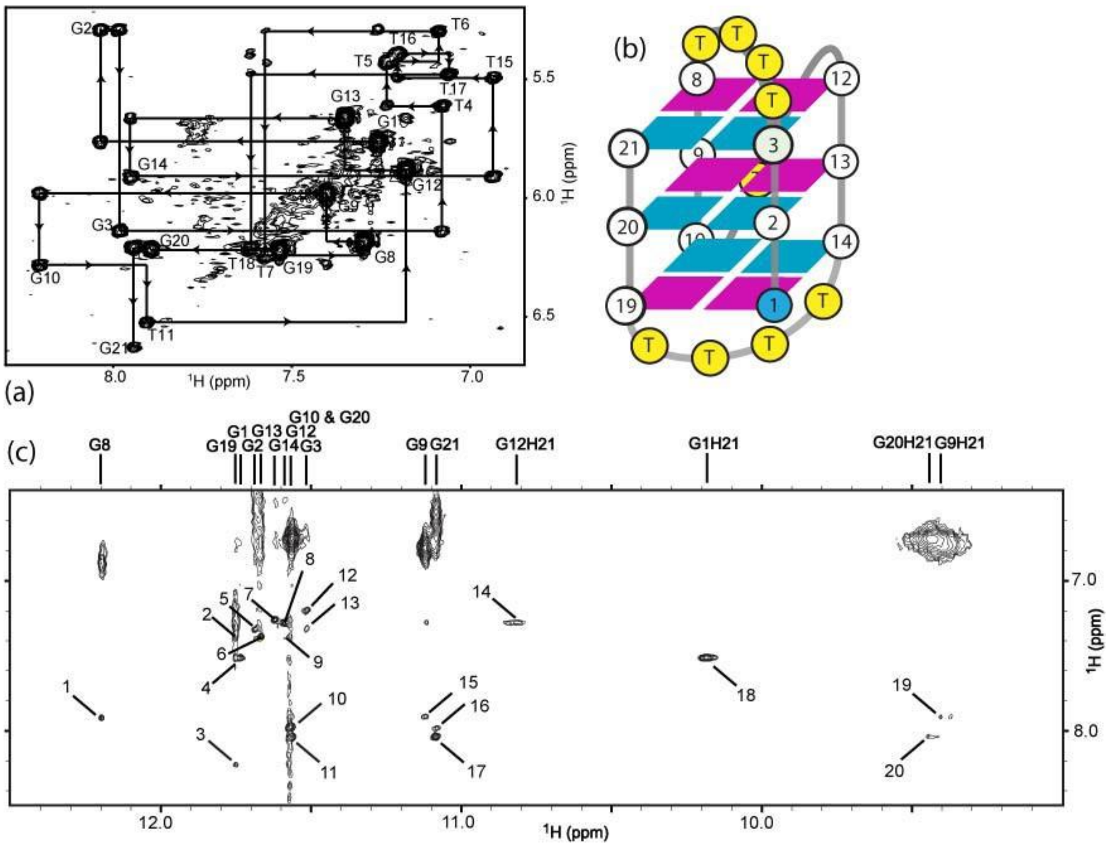

Fig. S2. NMR structure characterization of 2MFT. The DNA sequence d($G_3T_4G_3TG_3T_4G_3$) at 2 mM concentration adopts the 3(d+pd) topology in 16 mM NaCl, 4 mM NaH_2PO_4/Na_2HPO_4, pH 6.8 (PDB id 2MFT). Anomeric-aromatic at 25°C (A) and, aromatic-imino at 5°C (B) regions of the ^{1}H,^{1}H NOESY spectrum of 2MFT are shown, along with a schematic for the topology designed for this DNA sequence (C). In panel (A), intraresidual H1′-H6/H8 NOE interactions are labelled and sequential correlations are denoted with lines. In panel (B), H8-H1 cross-peaks within G1-G14-G10-G19, G2-G13-G9-G20, and G3-G12-G8- G21 tetrads are labeled. Peaks 1-20 are assigned as follows: (1) G8H1-G21H8, (2) G19H1-G9H8, (3) G19H1-G10H8, (4) G1H1-G19H8, (5) G2H1-G13H8, (6) G13H1-G9H8, (7) G14H1-G1H8, (8) G12H1-G8H8, (9) G12H1-G9H8, (10) G10H1-G14H8, (11) G20H1-G2H8, (12) G3H1-G12H8, (13) G3H1-G13H8, (14) G12H21-G8H8, (15) G9H1-G20H8, (16) G21H1-G3H8, (17) G21H1-G2H8, (18) G1H21-G19H8, (19) G9H21-G20H8 and (20) G20H21-G2H8. A complete list of proton assignments is shown in Table S1.

Structural assignments for 2M6W in sodium

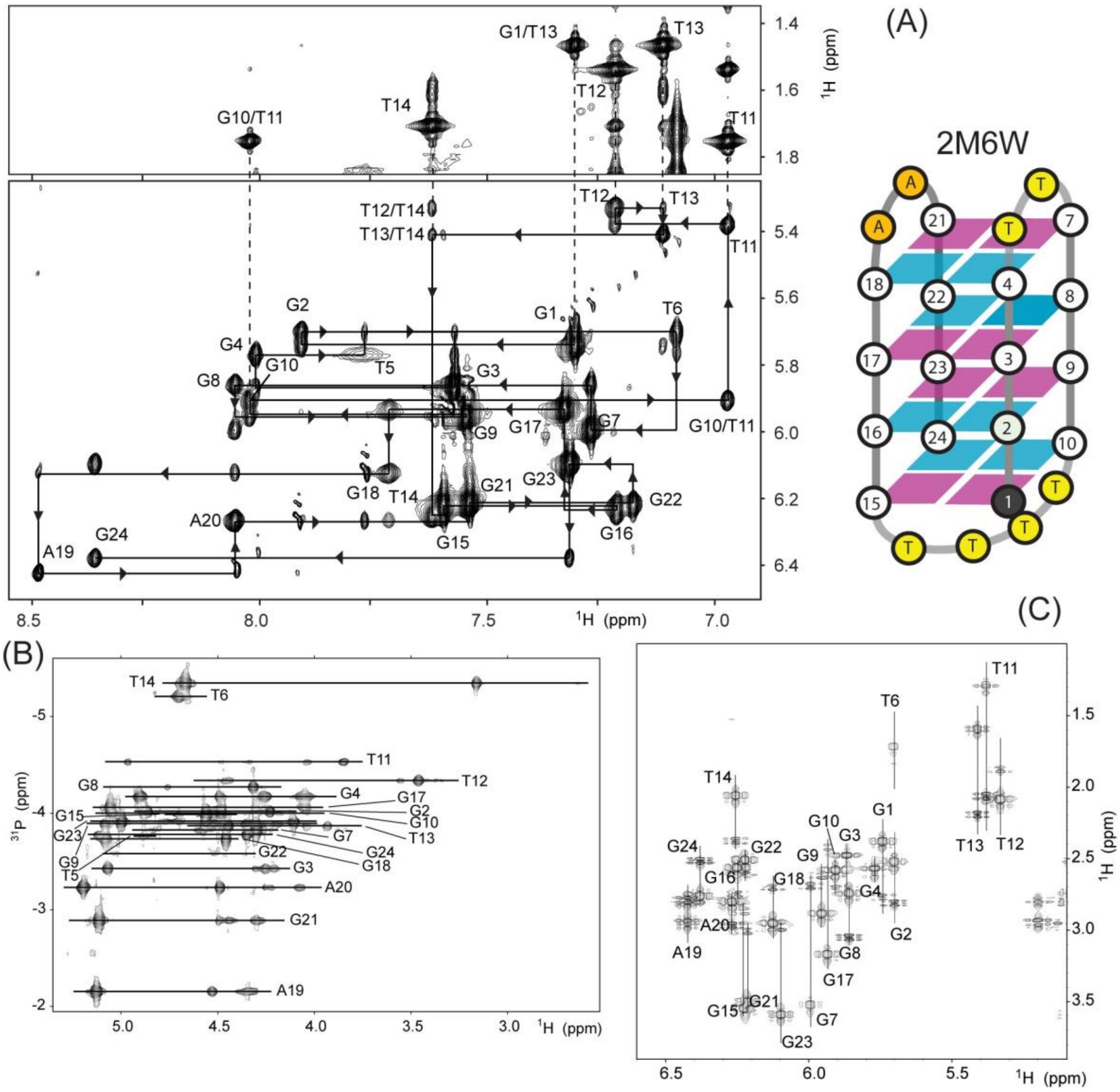

Fig. S3. NMR structure characterization of aromatic and anomeric regions of 2MW6. Non-exchangeable ^{1}H and ^{31}P assignments for the 4(-l_wd+l_n) adopted by the DNA sequence 2M6W in 16 mM NaCl, 4 mM NaH_2PO_4/Na_2HPO_4, pH 6.8, at 20 °C. In panel (A), expansions of ^{1}H- ^{1}H NOESY spectra (20 ^{o}C) depicting anomeric-aromatic regions of the ^{1}H-^{1}H NOESY and showing labelled intraresidual H1′-H6/H8 and H6/H8-H2’/2’’ NOE interactions. Sequential correlations are denoted with lines. The inset contains assignments for the characteristic sequential connectivities (*Syn*G-*Anti*G-T-T-T-T) of the diagonal loop. Methyl-H8/H6 sections illustrate the characteristic connectivity between the aromatic H8 of the 5’-*Syn*G residue of the stem and the methyl of the third Thymine in the diagonal loop. Shown are also schematic representations of the topologies they adopt with 2’-deoxyguanosines of the stem in *syn* (pink) and *anti* (cyan) conformations, green for non-stem guanosines, and yellow for thymines. In panel (B) sequential coupling correlations of the type H3’(i-1)-P(i)-H4’/H5’/H5’’ in a[^{1}H-^{31}P] HSQC spectrum are shown. In panel (C) DQF COSY intraresidue correlations H1’-H2’/H2’’ are shown. A complete list of proton assignments is shown in Table S2.

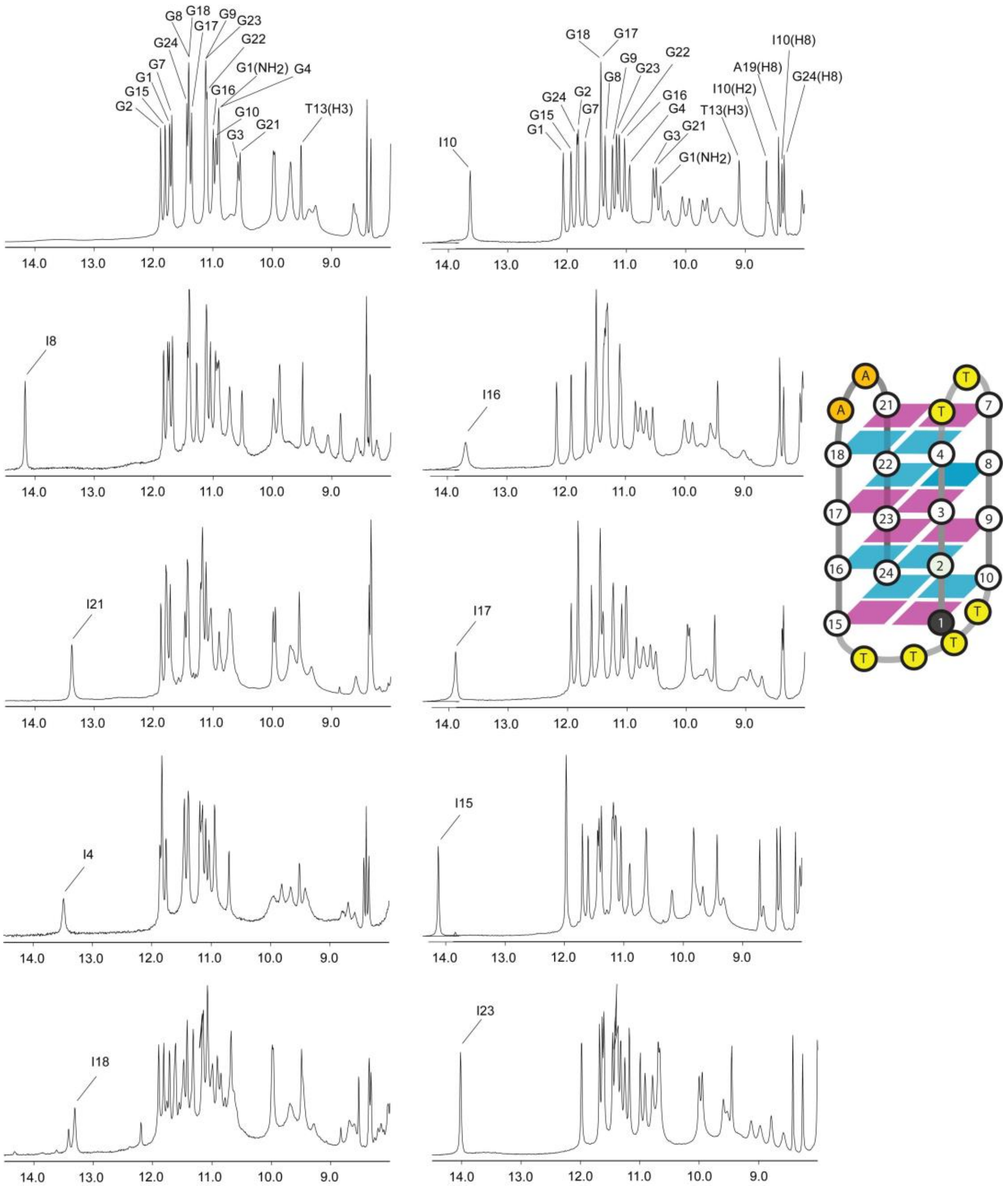

Fig. S4. NMR structure characterization of inosine substitutions for 2MW6. Exchangeable proton region expansions of NMR spectra of DNA sequence 2M6W (top left) and of its inosine substitutions (indicated) in sodium solutions at pH 6.8.

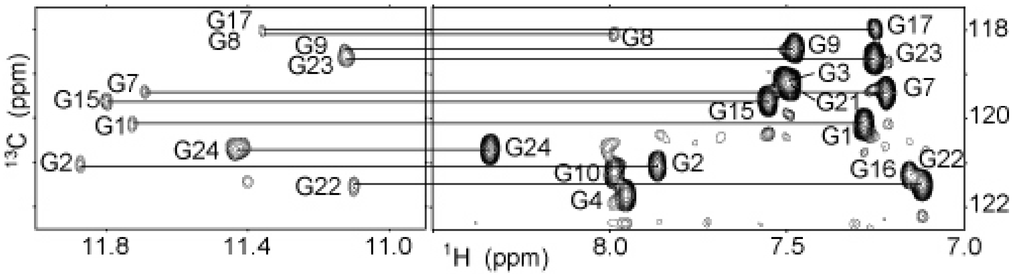

Fig. S5. Intraresidue aromatic-imino assignments for guanines in the stem of 2MW6. Heteronuclear natural abundance JR [^{1}H-^{13}C] H1(C5)H8 HMBC spectrum of 2M6W in 16 mM NaCl, 4 mM NaH_2PO_4/Na_2HPO_4, pH 6.8. Expansion illustrates the long range imino H1 to aromatic H8

coupling through the $^{13}C_5$ atom, in ^1H_2O at 15°C, providing unambiguous imino proton assignments for Guanines in the stem.

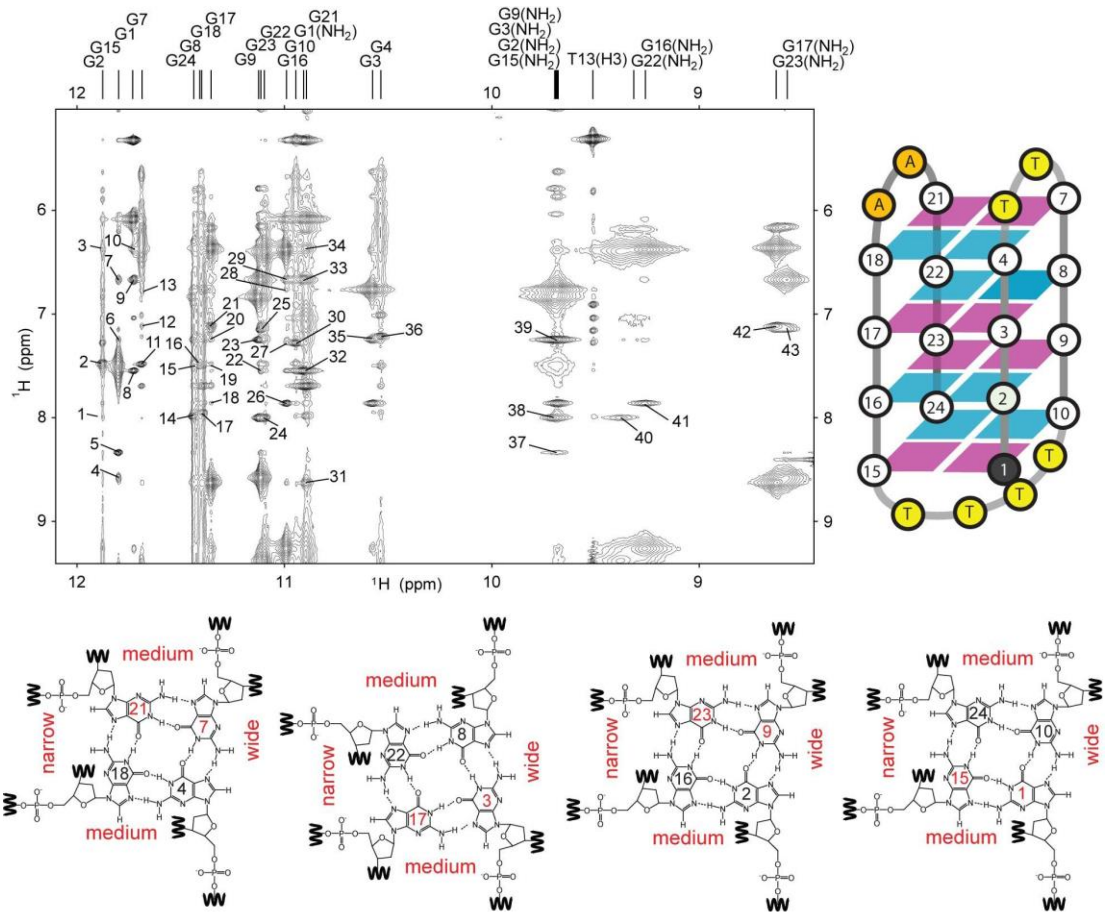

Fig. S6. Exchangeable proton assignments for the structure of 2M6W in 16 mM NaCl, 4 mM NaH_2PO_4/Na_2HPO_4, pH 6.8 at 5° C. An expansion of a JR-NOESY spectrum (250 ms) at ^2H_2O at 5 °C is shown, illustrating the dipolar connectivities between imino and amino exchangeable protons with aromatic protons. Peaks 1-43 are assigned as follows: (1) G2H1-G10H8, (2) G2H1-G9H8, (3) G2H1-G16H22, (4) G15H1-G23H21, (5) G15H1-G24H8, (6) G15H1-G23H8, (7) G15H1-G23H22, (8) G1H1-G15H8, (9) G1H1-G23H22, (10) G7H1-G21H8, (11) G7H1-G22H8, (12) G7H1-G3H22, (13) G24H1-G10H8, (14) G24H1-G15H22, (15) G8H1-G3H8, (16) G18H1-G4H8, (17) G17H1-G2H8, (18) G17H1-G21H8, (19) G17H1-G23H8, (20) G17H1-G22H8, (21) G23H1-G15H8, (22) G9H1-G23H8, (23) G22H1-G8H8, (24) G23H1-G16H8, (25) G16H1-G2H8, (26) G16H1-G1H8, (27) G16H1-G3H22, (28) G16H1-G23H22, (29) G10H1-G1H8, (30) G21H1-G17H21, (31) G1H21-G15H8, (32) G1H21-G23H22, (33) G21H1-G17H22, (34) G3H1-G17H8, (35) G4H1-G7H8, (36) G15H21-G24H8, (37) G2H21-G8H8, (38) G3H21-G17H8, (39) G22H21-G8H8, (40) G16H21-G2H8, (41) G17H21-G22H8, (43) G23H21-G16H8. These assignments allow for the formation of the hydrogen bond alignments shown in the chemical structures below defining the topology shown.

Structural assignments for 5J6U in sodium

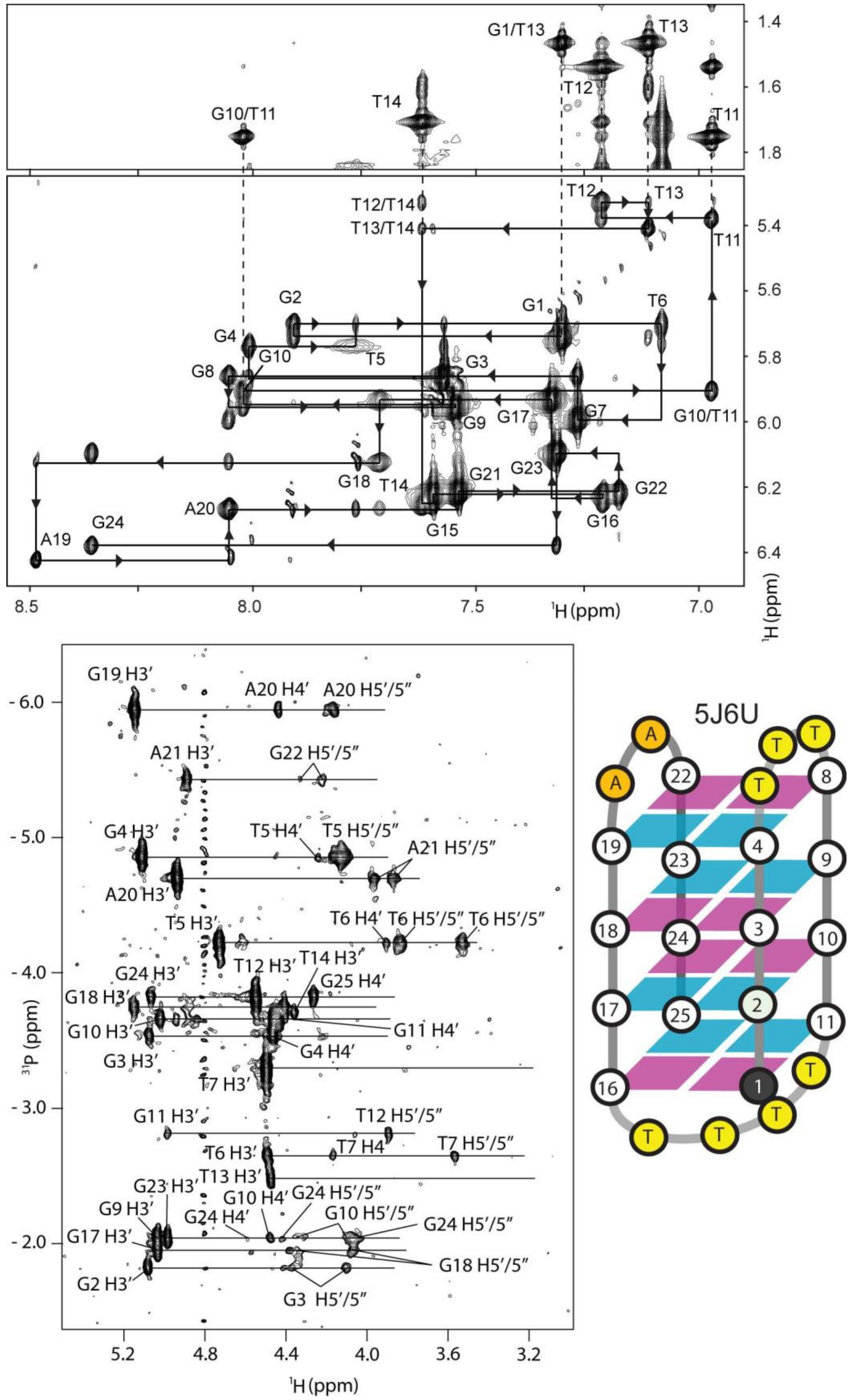

Fig. S7. Nonexchangeable ^{1}H and ^{31}P assignments for 5J6U. Non-exchangeable ^{1}H and ^{31}P assignments for the $4(-l_wd+l_n)$ adopted by the DNA sequence 5J6U in 16 mM NaCl, 4 mM NaH_2PO_4/Na_2HPO_4, pH 6.8, at 20 °C. The spectrum on top shows expansions of ^{1}H-^{1}H NOESY spectra (20 °C) depicting anomeric-aromatic regions of the ^{1}H-^{1}H NOESY and showing labelled

intraresidual H1′-H6/H8 and H6/H8-H2'/2'' NOE interactions. Sequential correlations are denoted with lines. The inset contains assignments for the characteristic sequential connectivities (*Syn*G-*Anti*G-T-T-T-T) of the diagonal loop. Methyl-H8/H6 sections illustrate the characteristic connectivity between the aromatic H8 of the 5'-*Syn*G residue of the stem and the methyl of the third Thymine in the diagonal loop. Shown are also schematic representations of the topologies they adopt with 2'-deoxyguanosines of the stem in *syn* (pink) and *anti* (cyan) conformations, orange for adenines, and yellow for thymines. In the bottom spectrum sequential coupling correlations of the type H3'(i-1)-P(i)-H4'/H5'/H5'' in a [^{1}H-^{31}P] HSQC spectrum are shown. A complete list of proton assignments is shown in Table S3.

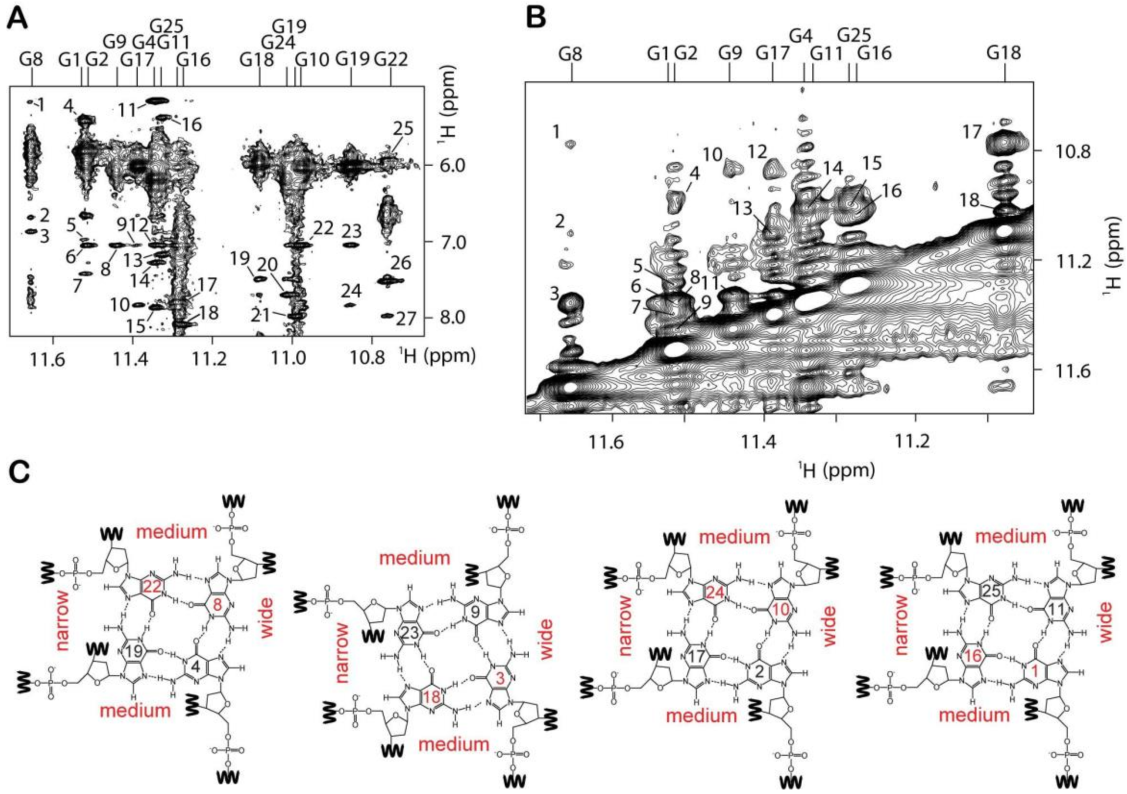

Fig. S8. Exchangeable proton assignments for 5J6U. In (A) an expansion of a JR-NOESY spectrum (240 ms) in 16 mM NaCl, 4 mM NaH_2PO_4/Na_2HPO_4, pH 6.8 at 5° C is shown, illustrating the dipolar connectivities between imino and amino exchangeable protons with aromatic protons. Peaks 1-27 are assigned as follows: (1) G8H1-T6H1', (2) G8H1-T7H8, (3) G8H1-G22H8, (4) G1H1-T14H1', (5)G1H1-T14H6, (6) G2H1-G10H8, (7) G1H1-G16H1, (8) G9H1-G3H8, (9) G17H1-G3H8, (10) G17H1-G2H8, (11) G4H1-T6H1', (12) G4H1-G3H1, (13) G11H1-G1H1, (14) G4H1-G8H8, (15) G4H1-G9H8, (16) G11H1-T12H1', (17) G25H1-G11H8, (18) G16H1-G25H1, (19) G18H1-G23H8, (20) G24H1-G17H8, (21) G19H1-G4H8, (22) G10H1-G24H8, (23) G3H1-G18H8, (24) G3H1-G2H8, (25) G22H1-A21H1', (26) G22H1-A21H1', (27) G22H1-G19H8. In (B) exchangeable proton assignments for 4[T3,A2]. An expanded JR-NOESY spectrum (200 ms) in 16 mM NaCl, 4 mM NaH_2PO_4/Na_2HPO_4, pH 6.8 at 5 °C is shown, illustrating the dipolar connectivities between imino H1-H1 exchangeable protons. Peaks 1-18 are assigned as follows: (1) G8H1-G22H1, (2) G8H1-G18H1, (3) G8H1-G4H1, (4) G2H1-G10H1, (5) G1H1-G16H1, (6) G1H1-G11H1, (7) G1H1-G17H1, (8) G2H1-G11H1, (9) G2H1-G9H1, (10) G9H1-G3H1, (11) G9H1-G4H1, (12) G17H1-G3H1, (13) G17H1-G18H1, (14) G4H1-G19H1, (15) G25H1-G10H1, (16) G16H1-G24H1, (17) G18H1-G22H1, (18) G18H1-G24H1. These assignments allow for the formation of the hydrogen bond alignments shown in the chemical structures shown in (C).

Structural assignments for 5J05 in sodium

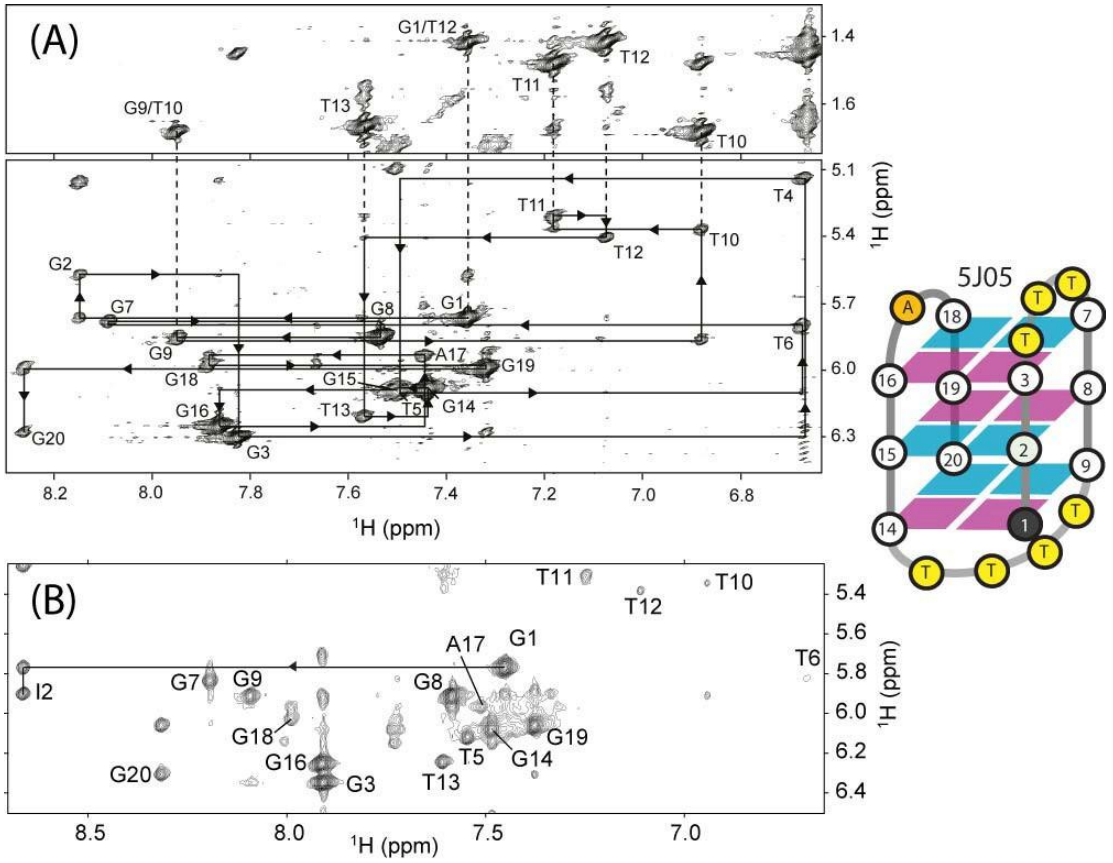

Fig. S9. Nonexchangeable ^{1}H assignments for 5J05. Non-exchangeable ^{1}H assignments for the $3(-l_wd+l_n)$ adopted by the DNA sequence 5J05 in 80 mM NaCl, 20 mM NaH_2PO_4/Na_2HPO_4, pH 6.8. In panel (A), expansions of ^{1}H-^{1}H NOESY spectra (20°C) depicting anomeric-aromatic regions of the ^{1}H-^{1}H NOESY and showing labelled intraresidual H1′-H6/H8 and H6/H8-H2'/2'' NOE interactions. Sequential correlations are denoted with lines. The inset contains assignments for the characteristic sequential connectivities (*Syn*G-*Anti*G-T-T-T-T) of the diagonal loop. Methyl-H8/H6 sections illustrate the characteristic connectivity between the aromatic H8 of the 5'-*Syn*G residue of the stem and the methyl of the third Thymine in the diagonal loop. Shown are also schematic representations of the topologies they adopt with 2'-deoxyguanosines of the stem in *syn* (pink) and *anti* (cyan) conformations, orange for adenines, and yellow for thymines. In panel (B) an expansion of JR [^{1}H-^{1}H] NOESY spectrum (200 ms mixing time) in ^1H_2O at 5°C of the substitution of G2 for Inosine in DNA sequence 5J05. The spectrum illustrates the chemical shift of H8 upon substitution, which unambiguously proves the assignment of the sequentially linked G1 as a *syn* residue for 5J05. A complete list of proton assignments is shown in Table S4.

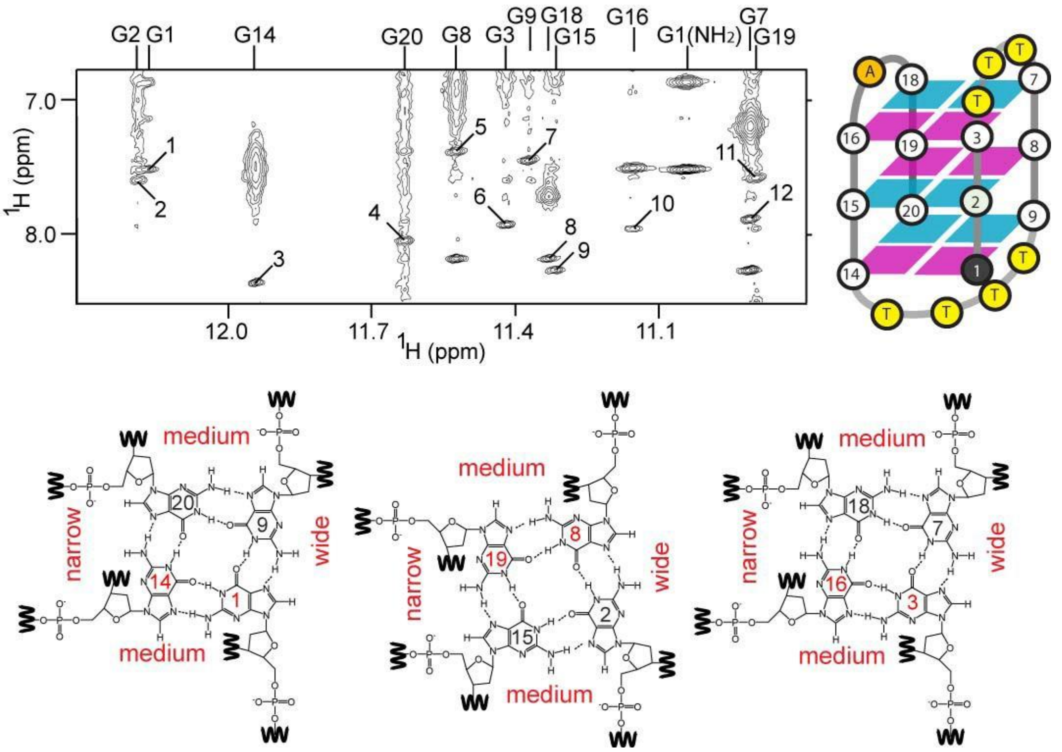

Fig. S10. Exchangeable proton assignments for 5J05 at 5 ^{o}C. An expansion of a JR-NOESY spectrum (200 ms) in 80 mM NaCl, 20 mM NaH_2PO_4/Na_2HPO_4, pH 6.8 is shown, illustrating the dipolar connectivities between imino H1-H8 imino H1-H1. Peaks 1-12 are assigned as follows: (1) G1H1-G14H8, (2) G2H1-G8H8, (3) G14H1-G20H8, (4) G20H1-G9H8, (5) G8H1-G19H8, (6) G3H1-G16H8, (7) G9H1-G1H8, (8) G18H1-G7H8, (9) G15H1-G2H8, (10) G16H1-G18H8, (11) G19H1-G15H8, (12) G7H1-G3H8. These assignments allow for the formation of the hydrogen bond alignments shown in the chemical structures below.

Structural assignments for 5J4W in sodium

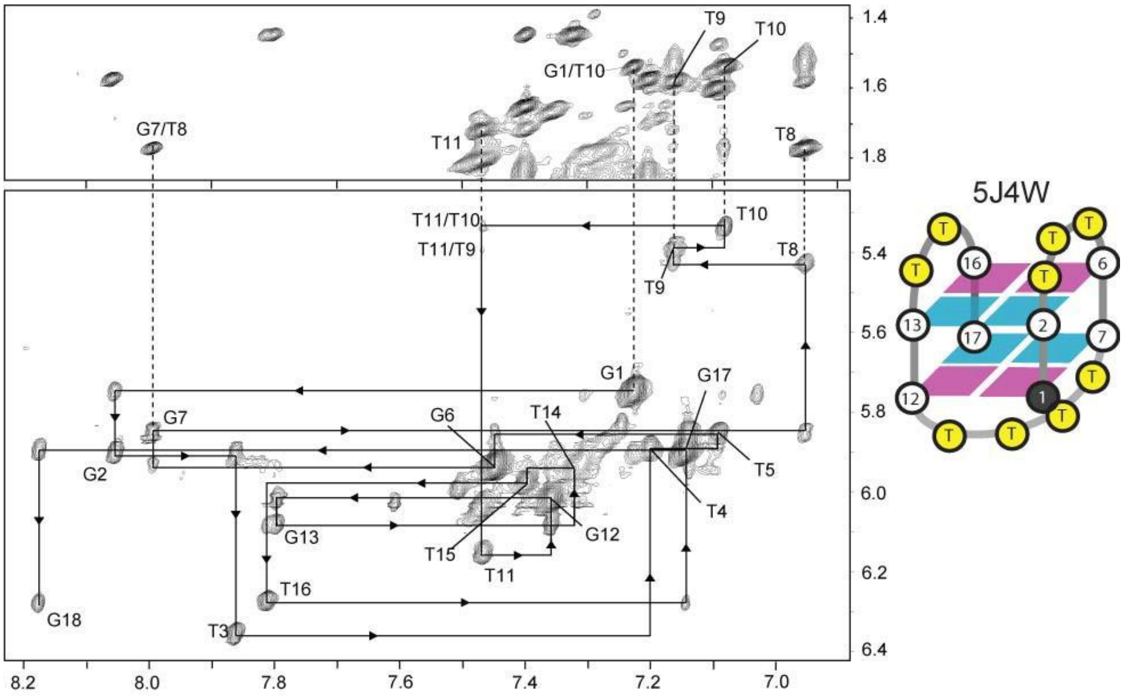

Fig. S11. Sequence-specific assignments for 5J4W. Non-exchangeable ^{1}H assignments for the 2(-l_wd+l_n) adopted by the DNA sequence 5J4W in 80 mM NaCl, 20 mM NaH_2PO_4/Na_2HPO_4, pH 6.8 and 20 ^oC. Expansions of ^{1}H-^{1}H NOESY spectra (20°C) depicting anomeric-aromatic regions of the ^{1}H-^{1}H NOESY and showing labelled intraresidual H1′-H6/H8 and H6/H8-H2'/2'' NOE interactions. Sequential correlations are denoted with lines. The inset contains assignments for the characteristic sequential connectivities (*Syn*G-*Anti*G-T-T-T-T) of the diagonal loop. Methyl-H8/H6 sections illustrate the characteristic connectivity between the aromatic H8 of the 5'-*Syn*G residue of the stem and the methyl of the third Thymine in the diagonal loop. Shown are also schematic representations of the topologies they adopt with 2'-deoxyguanosines of the stem in *syn* (pink) and *anti* (cyan) conformations, and yellow for thymines. A complete list of proton assignments is shown in Table S5.

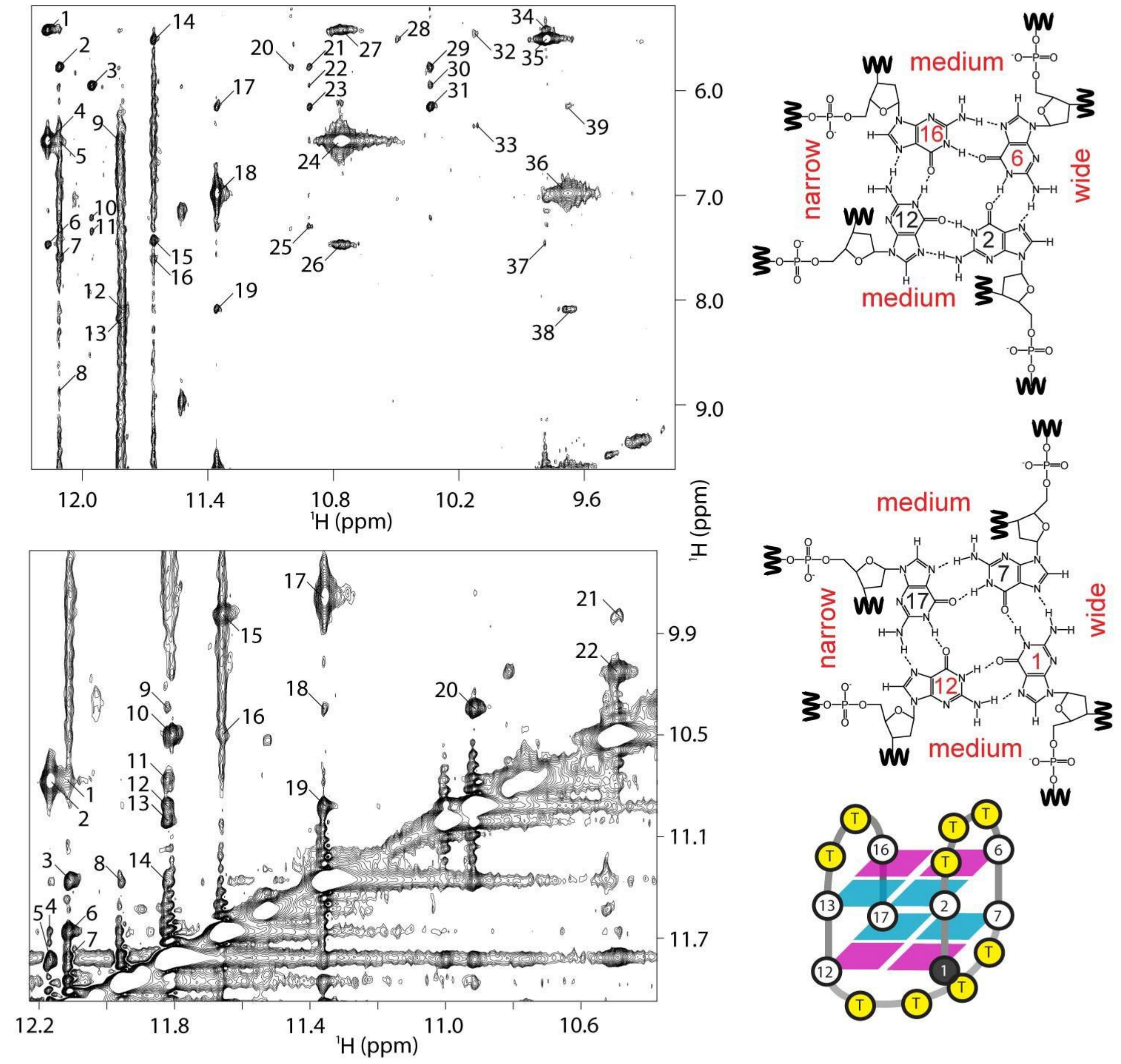

Fig. S12. Exchangeable proton assignments for 5J4W. Expansions of a JR-NOESY (200 ms) spectrum of the DNA sequence 5J4W in 80 mM NaCl, 20 mM Na_2HPO_4/NaH_2PO_4, pH 6.8 in ^1H_2O at 5 °C. Top spectrum: illustration of NOE connectivities between imino and aromatic protons. Peaks 1-39 are assigned as follows: (1) G1H1- T10H1', (2) G2H1-T4H1', (3) G6H1-T5H1', (4) G1H1-G1H22, (5) G12H1-G1H22, (6) G1H1-G12H8, (7) G2H1-G6H8, (8) G12H1-G17H8, (9) G13H1-G1H22, (10) G6H1-T5H6, (11) G6H1-G16H8, (12) G17H1-G7H8, (13) G13H1-G2H8, (14) G7H1-T8H1', (15) G7H1-G1H8, (16) G7H1-T11H6, (17) G16H1-T15H1', (18) G16H1-G16H22, (19) G16H1-G13H8, (20) T4H3-T4H1', (21) T15H3-T4H1', (22) T15H3-T5H1', (23) T15H3-T15H1', (24) G1H21-G1H22, (25) T15H3-T15H6, (26) G1H21-G12H8, (27) G1H21-T10H1', (28) T8H3-T8H1', (29) T5H3-T4H1', (30) T5H3-T5H1', (31) T5H3-T15H1', (32) T9H3-T9H1', (33) T9H3-T11H1', (34) T10H3-T10H1' (35) T10H3-T8H1' (36) G16H21-G16H22 (37) T10H3-G1H8, (38) G16H21-G13H8, (39) G16H21-T15H1'. Bottom spectrum: illustration of imino-aromatic and imino-imino proton connectivities. Peaks 1-22 are assigned as follows: (1) G12H1-G1H21, (2) G1H1-G1H21, (3) G12H1-G16H1, (4) G1H1-G7H1, (5) G1H1-G13H1, (6) G2H1-G7H1, (7) G2H1-G13H1, (8) G6H1-G16H1, (9) G13H1-T5H3, (10) G17H1-T8H3, (11) G13H1-G1H21, (12) G13H1-T15H3, (13) G13H1-T4H3, (14) G13H1-G16H1, (15) G7H1-T10H3, (16) G7H1-T8H3, (17) G16H1-G16H21, (18) G16H1-T5H3, (19) G16H1-T15H3, (20) T15H3-T5H3, (21) T8H3-T10H3, (22) T8H3-T9H3. These assignments allow for the formation of the hydrogen bond alignments depicted in the chemical structures shown.

Structural assignments for 5J4P in sodium

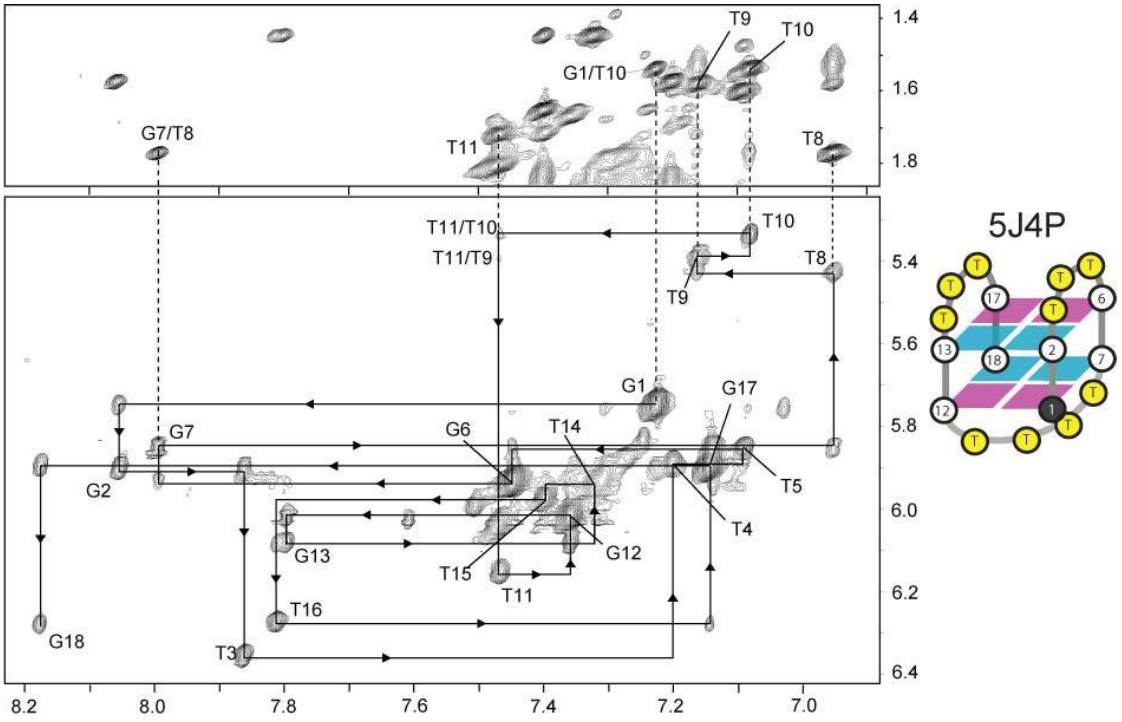

Fig. S13. Nonexchangeable ^{1}H assignments for 5J4P. Non-exchangeable ^{1}H assignments for the 2(-$l_w d+l_n$) adopted by the DNA sequence 5J4P in 80 mM NaCl, 20 mM NaH_2PO_4/Na_2HPO_4, pH 6.8 and 20 °C. Expansions of ^{1}H-^{1}H NOESY spectra depicting anomeric-aromatic regions of the ^{1}H-^{1}H NOESY and showing labelled intraresidual H1′-H6/H8 and H6/H8-H2’/2’’ NOE interactions. Sequential correlations are denoted with lines. The inset contains assignments for the characteristic sequential connectivities (*Syn*G-*Anti*G-T-T-T-T) of the diagonal loop. Methyl-H8/H6 sections illustrate the characteristic connectivity between the aromatic H8 of the 5’-*Syn*G residue of the stem and the methyl of the third Thymine in the diagonal loop. Shown are also schematic representations of the topologies they adopt with 2’-deoxyguanosines of the stem in *syn* (pink) and *anti* (cyan) conformations, and yellow for thymines. A complete list of proton assignments is shown in Table S6.

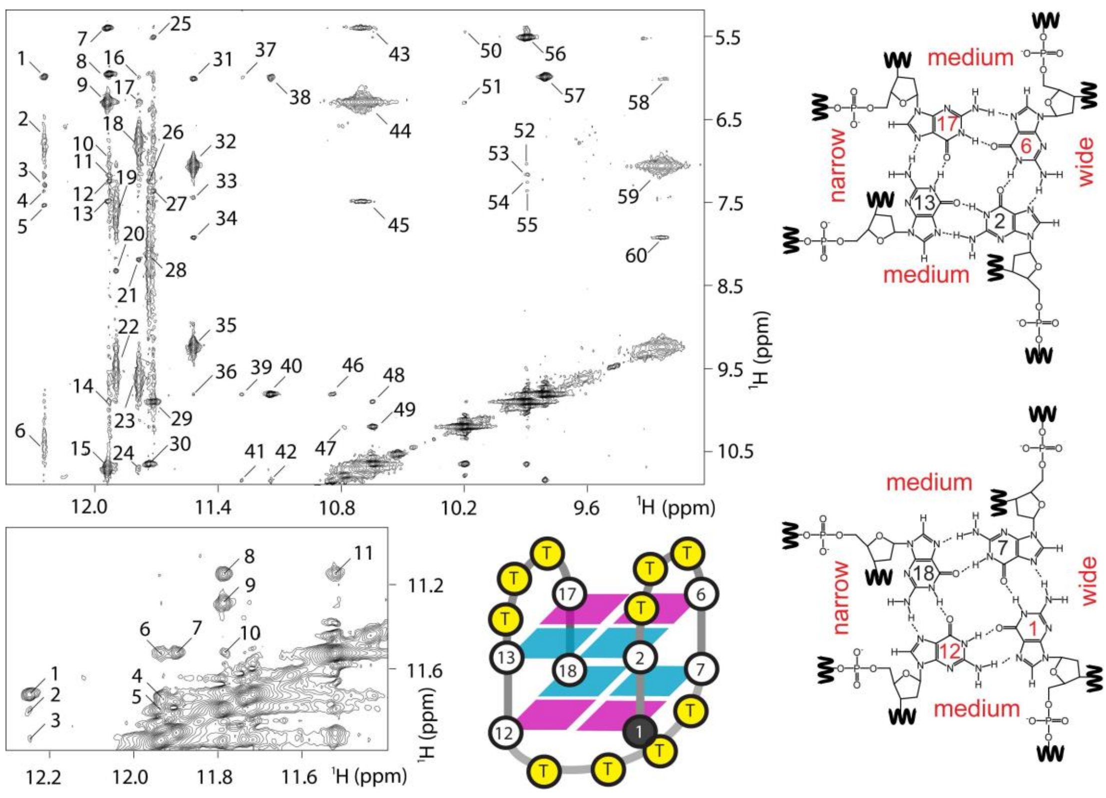

Fig. S14. Exchangeable proton assignments for 5J4P. Expansions of a JR-NOESY (200 ms) spectrum of 5J4P collected in 80 mM NaCl, 20 mM Na_2HPO_4/NaH_2PO_4, pH 6.8 in ^1H_2O at 5 °C. Top spectrum illustrates NOE connectivities between imino and aromatic protons. Peaks 1-60 are assigned as follows: (1) G2H1-G2H1', (2) G2H1-G2H22, (3) G2H1-T4H6, (4) G2H1-G1H8, (5) G2H1-G6H8, (6) G2H1-G2H21, (7) G6H1-G10H1', (8) G6H1-T5H1', (9) G1H1-G1H22, (10) G6H1-G6H22, (11) G6H1-T5H6, (12) G6H1-G17H8, (13) G1H1-G12H8, (14) G6H1-G6H21, (15) G1H1-G1H21, (16) G13H1-G2H1', (17) G13H1-G1H22, (18) G13H1-G13H22, (19) G12H1-G12H22, (20) G12H1-G18H8, (21) G13H1-G2H8, (22) G12H1-G12H21, (23) G13H1-G13H21, (24) G13H1-G1H21, (25) G7H1-T8H1', (26) G18H1-G17H8, (27) G7H1-G1H8, (28) G18H1-G7H8, (29) G7H1-T10H3, (30) G18H1-T8H3, (31) G17H1-G17H1', (32) G17H1-G17H22, (33) G17H1-T14H6, (34) G17H1-G13H8, (35) G17H1-G17H21, (36) G17H1-T16H3, (37) T15H3-G2H1', (38) T14H3-G2H1', (39) T15H3-T16H3, (40) T14H3-T16H3, (41) T15H3-T5H3, (42) T14H3-T5H3, (43) G1H21-T10H1', (44) G1H21-G1H22, (45) G1H21-G12H8, (46) T15H3-T16H3, (47) T11H3-T9H3, (48) T8H3-T10H3, (49) T8H3-T9H3, (50) T9H3-T9H1', (51) T9H3-T11H1', (52) T10H3-T8H6, (53) T10H3-T10H6, (54) T10H3-T9H6, (55) T10H3-G1H8, (56) T10H1-T8H1', (57) T16H1-T4H1', (58) G17H21-G17H1', (59) G17H21-G17H22, (60) G17H21-G13H8. Bottom spectrum illustrates imino to imino proton connectivities. Peaks 1-12 are assigned as follows: (1) G2H1-G7H1, (2) G2H1-G13H1, (3) G2H1-G6H1, (4) G6H1-G18H1, (5) G1H1-G17H1, (6) G6H1-G17H1, (7) G12H1-G17H1, (8) G13H1-T14H3, (9) G13H3-T15H3, (10) G13H1-G17H1, (11) G17H1-T14H3. These assignments allow for the formation of the hydrogen bond alignments depicted in the chemical structures shown.

Structural assignments for 2M6V in sodium

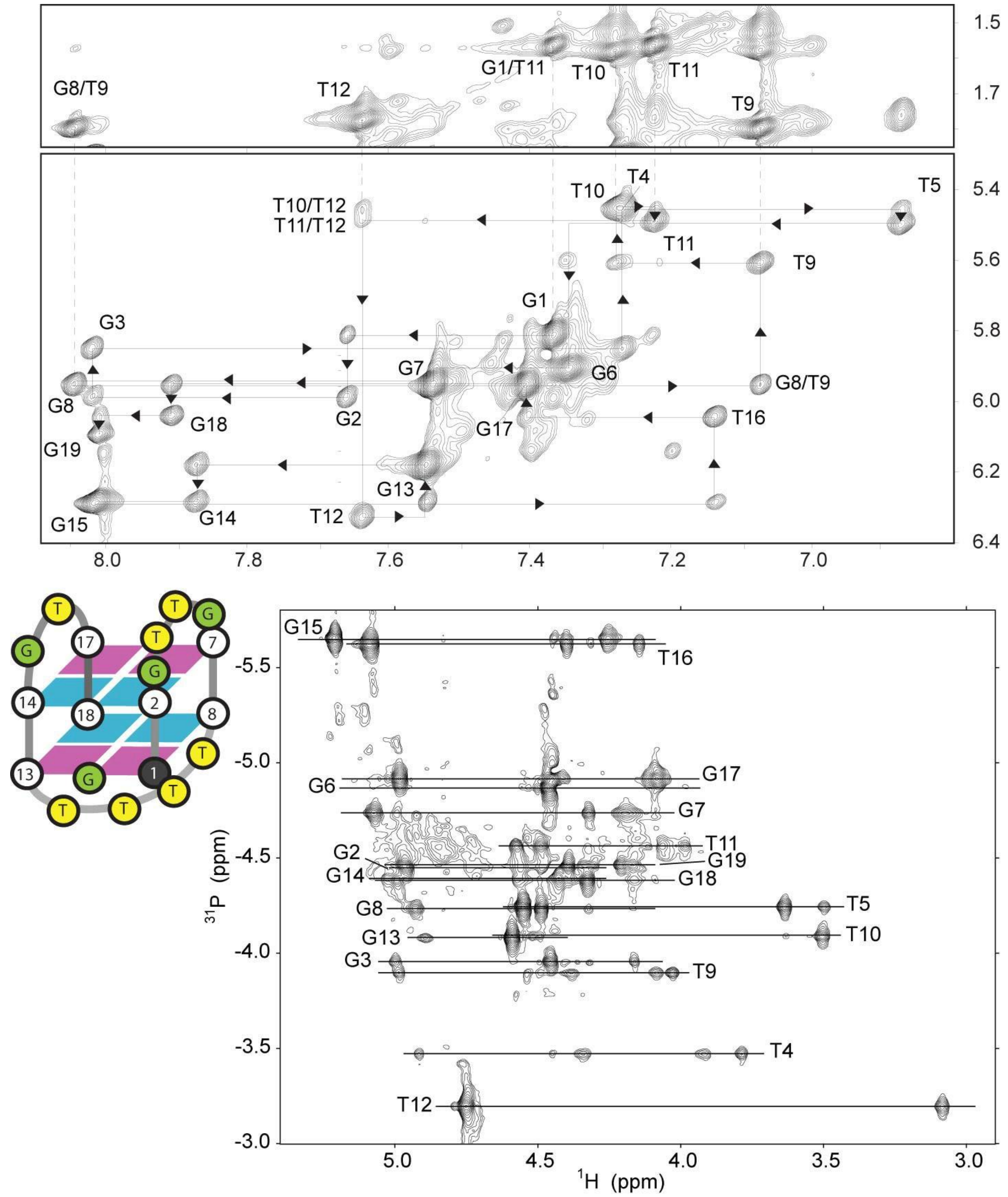

Fig. S15. Nonexchangeable ^{1}H and ^{31}P assignments for 2M6V. Non-exchangeable ^{1}H and ^{31}P assignments for the $2(-l_wd+l_n)$ adopted by the DNA sequence 2M6V in 20 mM NaCl, 4 mM NaH_2PO_4/Na_2HPO_4, pH 6.8, at 20 °C. The spectrum on top shows expansions of ^{1}H-^{1}H NOESY spectra (20 ^{o}C) depicting anomeric-aromatic regions of the ^{1}H-^{1}H NOESY and showing labelled intraresidual H1′-H6/H8 and H6/H8-H2'/2'' NOE interactions. Sequential correlations are denoted with lines. The inset contains assignments for the characteristic sequential connectivities (*Syn*G-*Anti*G-T-T-T-T) of the diagonal loop. Methyl-H8/H6 sections illustrate the characteristic connectivity between the aromatic H8 of the 5'-*Syn*G residue of the stem and the methyl of the third Thymine in the diagonal loop. Shown are also schematic representations of the topologies they adopt with 2'-deoxyguanosines of the stem in *syn* (pink) and *anti* (cyan) conformations, orange for adenines, and yellow for thymines. In the bottom spectrum sequential coupling correlations of the type H3'(i-1)-P(i)-H4'/H5'/H5'' in a [^{1}H-^{31}P] HSQC spectrum are shown. A complete list of proton assignments is shown in Table S7.

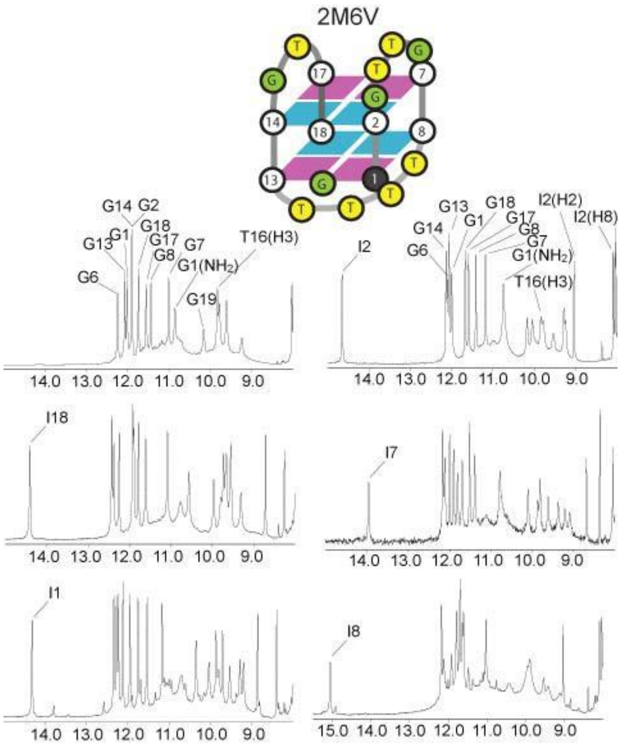

Fig. S16. Exchangeable proton perturbations for the inosine substitutions on 2M6V. The exchangeable proton region expansions of 1D ^{1}H JR spectra of sequence 2M6V and of its inosine substitutions in 20 mM NaCl, 4 mM NaH_2PO_4/Na_2HPO_4, pH 6.8 at 5 ^{o}C. On the right, the [^{1}H-^{31}P] HSQC correlation spectrum of 2M6V showing observed individual (for each residue) strips, including correlations of the type H3'(i-1)-P(i)-H4'/H5'/H5'' that were the basis of confirming sequence specific assignments and deriving angular restraints.

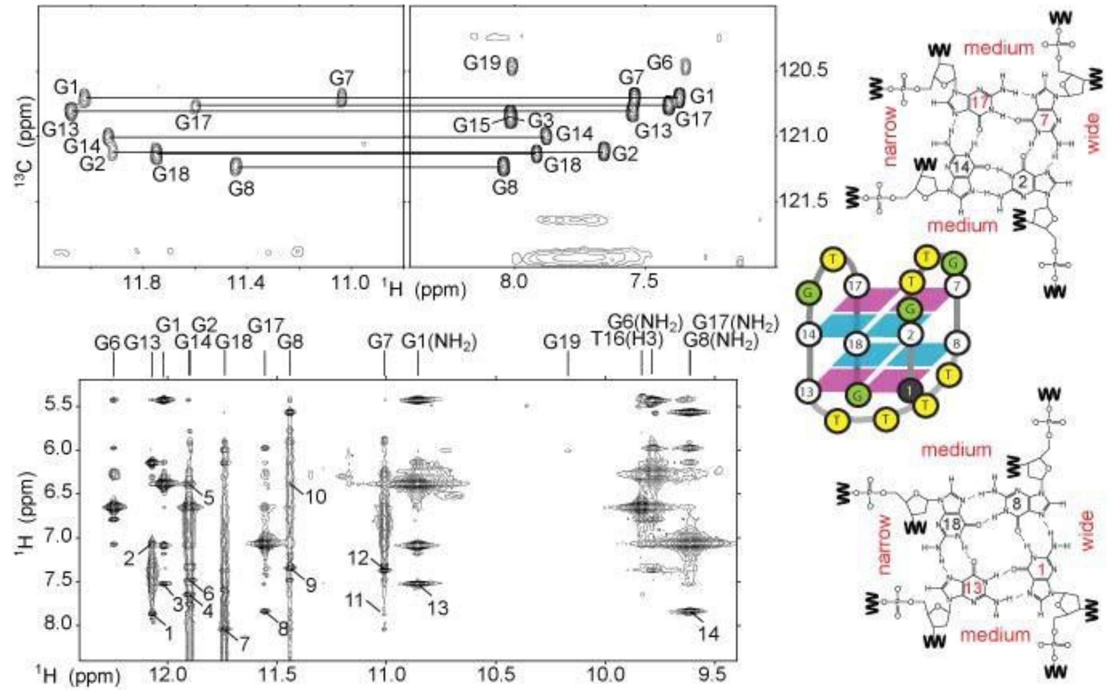

Fig. S17. Exchangeable proton assignments for 2M6V. Solution NMR experiments providing evidence for the topology formed by the DNA sequence 2M6V in 20 mM NaCl, 4 mM NaH_2PO_4/Na_2HPO_4, pH 6.8. Top spectrum shows an expansion of the natural abundance JR

[^{1}H-^{13}C] HMBC spectrum illustrating the long range imino H1 to aromatic H8 coupling through the $^{13}C_5$ atom, in $^{1}H_2O$ at 10 °C. The bottom spectrum shows the exchangeable proton region of a JR-NOESY spectrum (250 ms) at 5° C, illustrating the dipolar connectivities between imino and amino exchangeable protons with aromatic protons (A). Peaks 1-14 are assigned as follows: (1) G13H1-G18H8, (2) G13H1-G17H22, (3) G1H1-G13H8, (4) G14H1-G2H8, (5) G14H1-G1H22, (6) G2H1-G7H8, (7) G18H1-G8H8, (8) G17H1-G14H8, (9) G8H1-G1H8 (10), G8H1-G1H22, (11) G7H1-G18H8, (12) G7H1-G17H8, (13) G1H21-G13H8 and (14) G17H21-G14H8. These assignments allow for the formation of the hydrogen bond alignments depicted in the chemical structures shown.

Identification of topology

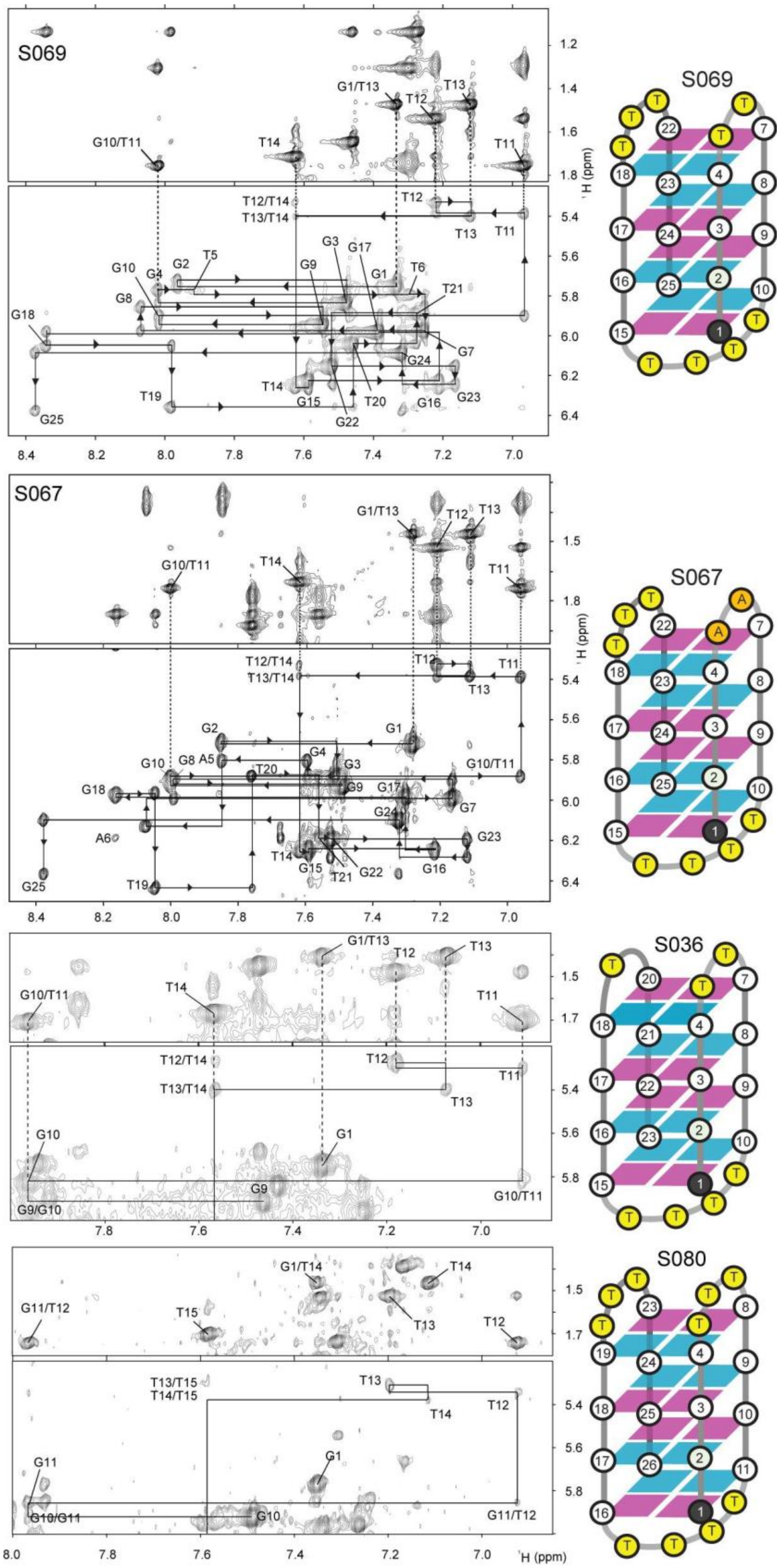

Fig. S18. NMR experiments for characterization of the $4(-l_{w}d+l_{n})$ topology formed by the DNA sequences S069, S067, S036, and S080. Anomeric-aromatic regions of the ^{1}H-^{1}H NOESY (tm 250 ms, 20 ^{o}C) spectra of DNA sequences S069, S067, S036 and S080 in 80 mM NaCl, 20 mM NaH_2PO_4/Na_2HPO_4, pH 6.8. Intraresidual H1′-H6/H8 and H6/H8-H2’/2’’ NOE interactions are labelled and sequential correlations are denoted with lines. The inset contains assignments for the characteristic sequential connectivities (*Syn*G-*Anti*G-T-T-T-T) of the diagonal loop. Methyl-H8/H6

sections illustrate the characteristic connectivity between the aromatic H8 of the 5'-*Syn*G residue of the stem and the methyl of the third Thymine in the diagonal loop. Shown are also schematic representations of the topologies they adopt with 2'-deoxyguanosines of the stem in *syn* (magenta) and *anti* (cyan) conformations.

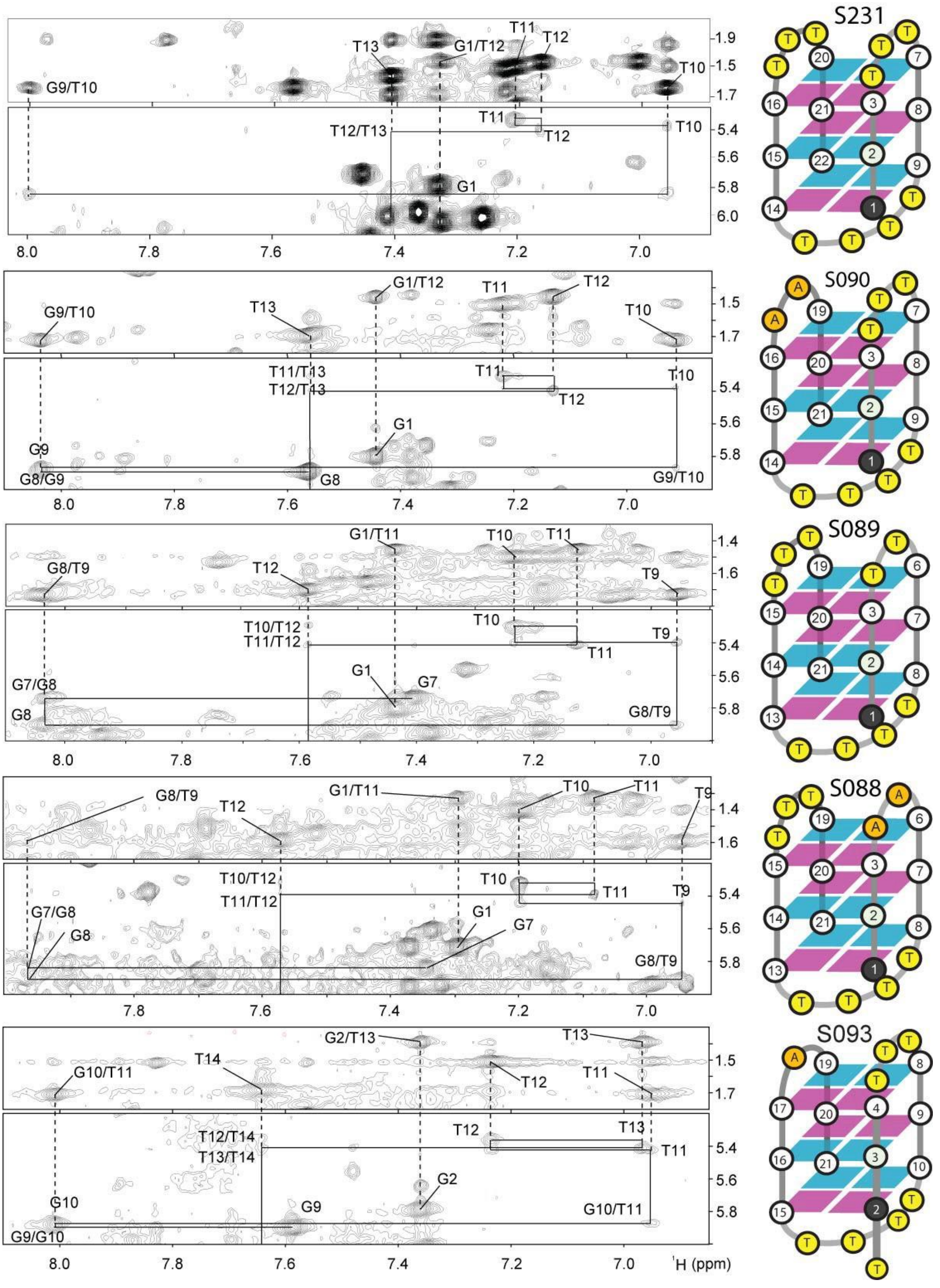

Fig. S19. Solution NMR experiments for characterization of the $3(-l_wd+l_n)$ topology formed by the DNA sequences S231, S090, S089, S088, and S093. Expansions of the anomeric-aromatic regions of the ^1H-^1H NOESY (mixing time 250 ms, 20°C) spectra of DNA sequences S231, S090, S089, S088, and S093 in 80 mM NaCl, 20 mM NaH_2PO_4/Na_2HPO_4, pH 6.8. Intraresidual H1′-H6/H8 NOE interactions are labelled and sequential correlations are denoted with lines. The inset contains

assignments for the characteristic sequential connectivities (*Syn*G-*Anti*G-T-T-T-T) of the diagonal loop. Methyl-H8/H6 sections illustrate the characteristic connectivity between the aromatic H8 of the 5'-*Syn*G residue of the stem and the methyl of the third Thymine in the diagonal loop. Shown are also schematic representations of the topologies they adopt with 2'-deoxyguanosines of the stem in *syn* (magenta) and *anti* (cyan) conformations.

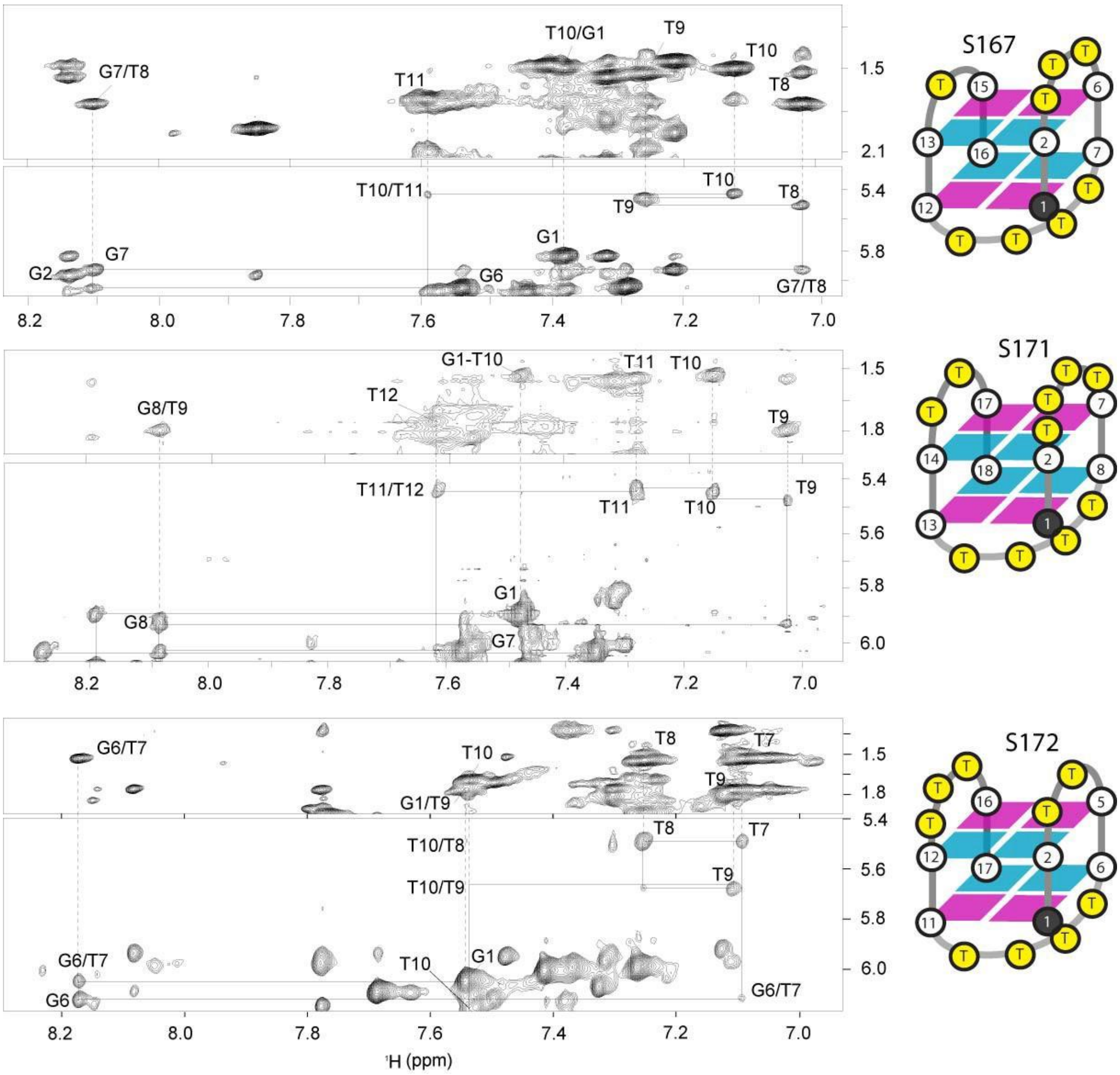

Fig. S20. Solution NMR experiments for characterization of the $2(-l_w d+l_n)$ topology formed by the DNA sequences S167, S171, and S172. Anomeric-aromatic regions of the ^{1}H-^{1}H NOESY (tm 250 ms, 20 ^{o}C) spectra of DNA sequences S167, S171, and S172 in 80 mM NaCl, 20 mM NaH_2PO_4/Na_2HPO_4, pH 6.8. Intraresidual H1′-H6/H8 NOE interactions are labelled and sequential correlations are denoted with lines. The inset contains assignments for the characteristic sequential connectivities (*Syn*G-*Anti*G-T-T-T-T) of the diagonal loop. Methyl-H8/H6 sections illustrate the characteristic connectivity between the aromatic H8 of the 5'-*Syn*G residue of the stem and the methyl of the third thymine in the diagonal loop. Shown are also schematic representations of the topologies they adopt with 2'-deoxyguanosines of the stem in *syn* (magenta) and *anti* (cyan) conformations.

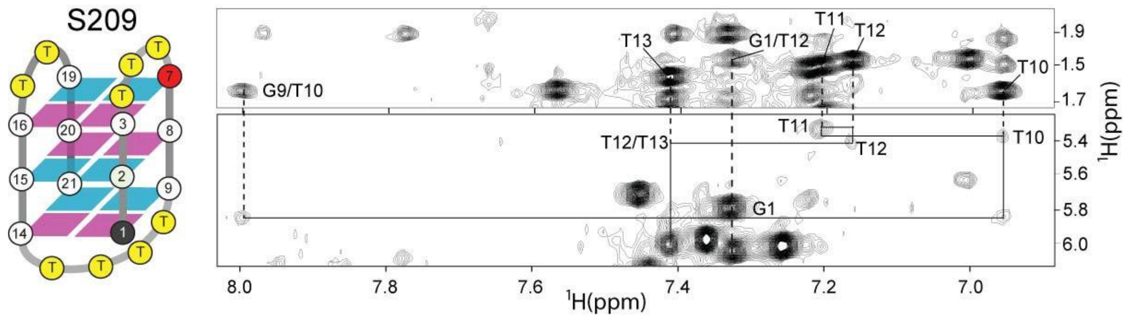

Fig. S21. Use of riboguanosines to induce folding of the 3(−l_wd+l_n) topology. Schematics of the topology adopted by S209 in 80 mM NaCl, 20 mM NaH_2PO_4/Na_2HPO_4, pH 6.8. Guanosines in tetrads comprising the quadruplex stem are displayed as pink (*syn*G) and blue (*anti*G) squares, with numbered white (deoxyriboguanosine) or red (riboguanosine) circles indicating their position in the primary sequence. Thymines of each loop are shown as yellow circles. Expansions of proton NOESY spectrum at 250 ms mixing time and 20 °C, illustrating the presence of the characteristic sequential connectivities (*syn*G-*anti*G-T-T-T-T) of the diagonal loop in the 3(-l_wd+l_n) topology. For each sequential assignment the characteristic connectivity between the aromatic H8 of the 5’-*Syn*G residue of the stem and the methyl of the third Thymine in the diagonal loop is illustrated.

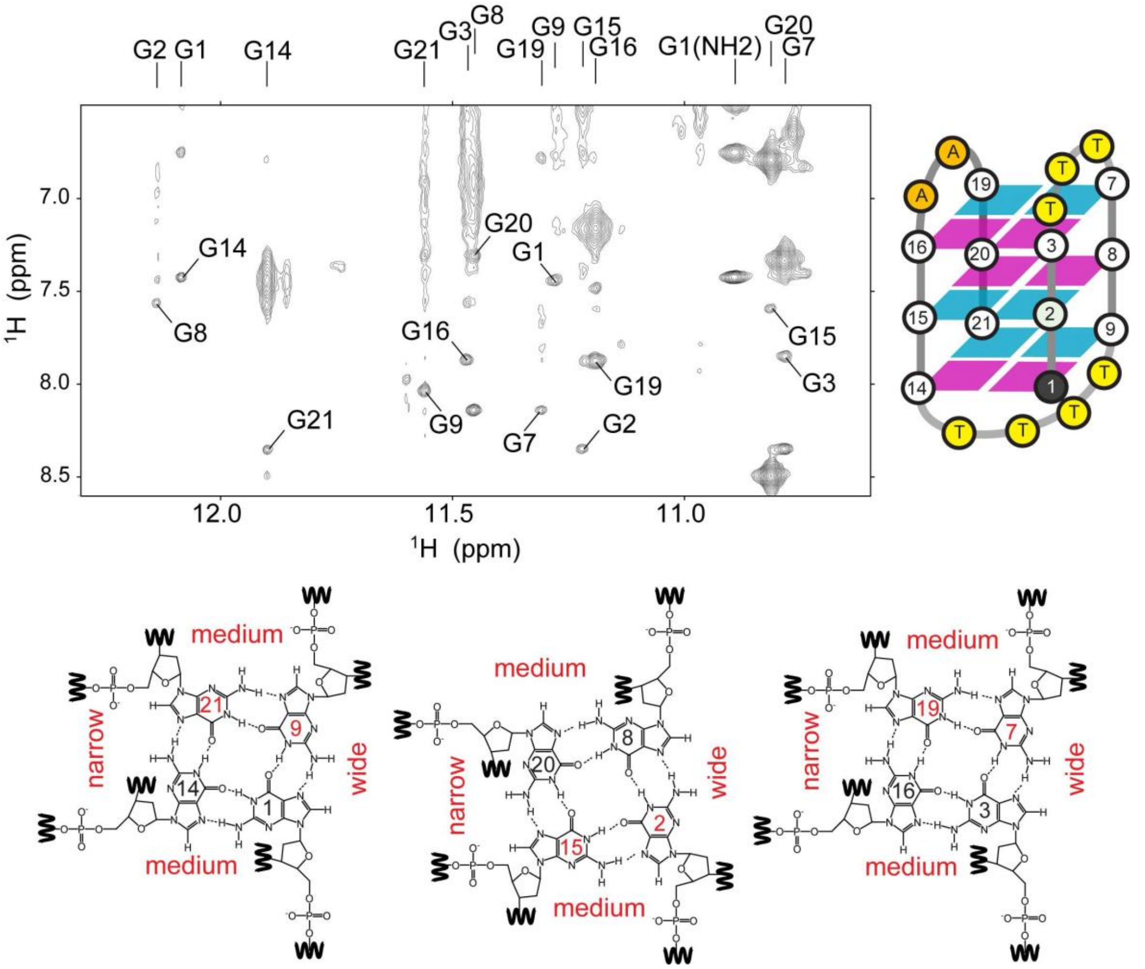

Fig. S22. Exchangeable proton assignments for 3(−l_wd+l_n) topology formed by S090. Exchangeable proton assignments for S090 in 80 mM NaCl, 20 mM NaH_2PO_4/Na_2HPO_4, pH 6.8. An expanded [^{1}H-^{1}H] JR-NOESY spectrum (200 ms) of S090 at 5° C, depicts NOE connectivities between imino (H1) exchangeable protons and aromatic (H8) protons. Sequential connectivities and those with loop residues are not indicated. These assignments allow for the hydrogen bond alignments depicted in the chemical structures below defining the topology shown.

NMR chemical shifts tables

Table S1. Proton chemical shifts for the structure of 2MFT. Exchangeable-proton chemical shifts were obtained at 5° C while non-exchangeable proton chemical shifts were obtained at 20° C. H5’/H5’’ protons are not stereo-specifically assigned.

Residue	H1/H3	H21	H22	H8/H6	CH_3	H1’	H2’	H2’’	H3’	H4’	H5’	H5’’
G1	11.736	10.184	6.215	7.253	-	5.766	2.894		5.064	4.308	4.972	4.014
G2	11.686	11.514	6.109	8.029	-	5.295	2.514	2.294	5.031	4.146	-	-
G3	11.514	-	-	7.976	-	6.141	2.820	2.581	4.940	4.367	4.063	
T4	-	-	-	7.073	1.753	5.614	2.274	1.509	4.503	4.007	-	-
T5	-	-	-	7.227	1.557	5.431	1.973	1.842	4.500	3.462	3.601	
T6	9.841	-	-	7.082	1.473	5.300	2.290	1.703	4.668	4.359	3.984	3.883
T7	-	-	-	7.566	1.660	6.236	2.367	2.073	4.524	3.183	3.014	2.790
G8	12.197	8.842	6.857	7.292	-	6.185	3.382	3.020	4.949	4.448	4.144	4.012
G9	11.119	9.395	6.776	7.395	-	5.982	3.227	2.864	5.020	4.443	4.317	
G10	11.574	-	-	8.199		6.284	2.663	2.447	4.766	4.298	4.213	
T11	-	-	-	7.900	2.003	6.526	2.793	2.422	5.072	4.382	-	-
G12	11.590	10.822	5.968	7.175	-	5.886	3.298		4.971	4.306	3.927	3.834
G13	11.669	-	-	7.346	-	5.667	2.508		5.034	4.140	-	-
G14	11.619	-	-	7.944	-	5.911	2.685	2.511	4.929	4.439	-	-
T15	-	-	-	6.931	1.685	5.494	2.143	1.365	4.465	3.898	4.322	4.036
T16	-	-	-	7.197	1.536	5.397	2.001	1.859	4.508	3.364	3.541	3.478
T17	9.896	-	-	7.055	1.425	5.481	2.297	1.672	4.655	4.479	4.035	3.934
T18	-	-	-	7.610	1.686	6.214	2.362	2.080	4.538	3.175	2.870	2.773
G19	11.571	9.549	7.299	7.528	-	6.220	3.579	3.033	4.930	4.728	4.138	
G20	11.565	9.434	6.722	7.888	-	6.211	2.923	2.565	5.087	4.585	4.385	4.288
G21	11.086	9.082	6.615	7.935	-	6.628	2.827	2.736	5.174	4.517	4.378	

Table S2. Proton and phosphorous chemical shifts for structure of 2M6W. Proton and phosphorous (20 °C) chemical shifts for sequence 2M6W in 20 mM NaPi (pH 6.8) aqueous buffer. Exchangeable-proton chemical shifts were obtained at 5° C while non-exchangeable proton chemical shifts were obtained at 20° C. H5'/H5'' protons are not stereo- specifically assigned.

Residue	H1/H3	H21	H22	P	H8/H6	H2	CH_3	H1'	H2'	H2''	H3'	H4'	H5'	H5''
G1	11.73	10.9	6.087	-	7.308	-	-	5.738	2.388	2.785	4.858	4.281	3.91	3.789
G2	11.88	9.691	-	-4.016	7.908	-	-	5.698	2.531	2.812	5.071	4.227	4.127	4.148
G3	10.58	9.685	6.766	-3.425	7.572	-	-	5.863	2.745	2.482	4.903	4.257	4.053	
G4	10.9	9.314	7.046	-4.171	8.008	-	-	5.768	2.568		4.882	4.49	4.062	
T5	-	-	-	-3.762	7.770	-	2.008	5.767	2.31	1.866	4.699	4.315	4.27	4.042
T6	-	-	-	-5.209	7.084	-	1.215	5.699	1.722	1.856	4.298	2.369	3.595	3.301
G7	11.69	10.06	6.305	-3.824	7.272	-	-	5.99	3.532	2.707	4.758	4.589	4.283	3.865
G8	11.41	-	-	-4.27	8.054	-	-	5.857	2.751	3.053	5.084	4.318	4.202	
G9	11.13	9.699	6.834	-3.889	7.546	-	-	5.951	2.891	2.625	5.04	4.354	4.192	
G10	10.95	9.955	-	-3.996	8.021	-	-	5.903	2.588	2.713	4.968	4.485	4.038	
T11	-	-	-	-4.529	6.971	-	1.751	5.377	1.300	2.071	4.45	3.851	3.79	3.931
T12	-	-	-	-4.337	7.219	-	1.538	5.325	2.089	1.886	4.539	3.463	3.554	3.391
T13	9.512	-	-	-3.866	7.113	-	1.465	5.407	1.602	2.202	4.669	4.447	4.069	3.932
T14	-	-	-	-5.343	7.621	-	1.706	6.252	2.066	2.381	4.558	3.162	2.639	2.879
G15	11.8	9.696	7.509	-3.984	7.597	-	-	6.22	3.557	2.878	4.943	4.717	4.496	4.142
G16	10.99	9.263	6.378	-	7.215	-	-	6.232	2.572	2.753	5.057	4.516	4.328	
G17	11.36	8.626	6.364	-4.057	7.33	-	-	5.933	3.176	2.433	5.08	4.485	4.056	4.325
G18	11.4	9.684	-	-3.73	7.717	-	-	6.122	2.959	2.714	5.124	4.457	4.109	4.252
A19	-	-	-	-2.153	8.486	7.766	-	6.419	2.951	2.801	5.196	4.529	4.367	4.336
A20	-	-	-	-3.229	8.054	7.912	-	6.263	2.971	2.808	5.115	4.488	4.078	4.241
G21	10.54	9.685	6.182	-2.887	7.539	-	-	6.211	3.514	3.018	5.031	4.443	4.493	4.298
G22	11.1	9.38	6.397	-3.579	7.179	-	-	6.214	2.520	2.616	4.998	4.519	4.366	
G23	11.11	8.577	6.668	-3.915	7.32	-	-	6.095	3.600	2.993	5.119	4.261	4.573	4.73
G24	11.44	9.697	6.835	-3.777	8.361	-	-	6.376	2.768	2.527	4.839	4.349	4.106	4.263

Table S3. Proton and phosphorous chemical shifts for structure of 5J6U. Proton and phosphorous (20 °C) chemical shifts for sequence 5J6U in 20 mM NaPi (pH 6.8) aqueous buffer. Exchangeable-proton chemical shifts were obtained at 5° C at 500 MHz, while non-exchangeable proton chemical shifts were obtained at 20° C at 900 MHz. H5'/H5'' protons are not stereo-specifically assigned.

Residue	H1/H3	H21	H22	P	H8/H6	H2	CH_3	H1'	H2'	H2''	H3'	H4'	H5'	H5''
G1	11.52	10.19	5.82	-	7.30	-	-	6.03	3.08	2.83	4.95	4.43	3.88	3.89
G2	11.51	9.25	6.03	-2.23	7.97	-	-	6.19	2.84	2.70	5.09	4.47	4.12	4.41
G3	10.87	8.92	-	-3.55	7.24	-	-	5.96	2.82	3.21	5.08	4.35	4.39	4.11
G4	11.35	9.60	6.16	-5.81	8.13	-	-	6.10	2.52	2.52	2.80	5.12	4.47	4.22
T5	-	-	-	-4.25	7.34	-	1.91	6.28	2.39	2.51	4.73	4.25	4.15	4.12
T6	-	-	-	-2.66	6.53	-	0.99	5.36	2.21	1.45	4.50	3.91	3.54	3.84
T7	-	-	-	-3.41	6.90	-	0.83	6.03	2.17	1.82	4.55	4.17	3.57	2.76
G8	11.66	9.46	5.86	-	7.46	-	-	6.00	3.14	2.96	4.72	4.39	4.32	4.17
G9	11.45	9.69	6.08	-2.06	7.99	-	-	6.17	2.79	2.63	5.04	4.48	4.31	4.09
G10	10.99	8.74	6.03	-3.62	7.24	-	-	5.9	2.65	3.04	5.03	4.49	4.10	4.32
G11	11.34	-	-	-2.86	7.91	-	-	5.97	2.68	2.53	4.99	4.44	4.18	4.13
T12	-	-	-	-3.76	7.10	-	1.78	5.6	2.12	1.82	4.62	4.33	3.90	4.06
T13	-	-	-	-2.48	7.32	-	1.67	5.67	2.03	1.88	4.50	3.70	-	-
T14	-	-	-	-3.66	7.18	-	1.41	5.73	2.04	1.82	-	4.35	4.34	3.82
T15	-	-	-	-	7.21	-	1.67	6.06	2.43	1.87	-	-	-	-
G16	11.28	9.32	7.22	-	7.59	-	-	6.14	3.04	3.50	4.85	4.64	4.12	4.11
G17	11.39	9.39	6.02	-1.92	7.87	-	-	6.16	2.71	2.57	5.04	4.46	4.23	4.08
G18	11.09	8.43	5.97	-3.70	7.23	-	-	6.03	3.30	2.88	5.16	4.57	4.36	4.07
G19	11.00	-	-	-5.96	8.13	-	-	6.2	2.62	2.80	5.15	4.94	4.40	4.42
A20	-	-	-	-4.64	8.24	8.10	-	6.32	2.54	2.70	4.94	4.44	4.18	4.16
A21	-	-	-	-5.43	7.68	7.64	-	6.12	2.51	3.02	4.89	4.34	3.97	3.87
G22	10.77	9.34	6.63	-	7.08	-	-	5.73	2.84	3.02	4.89	4.36	4.33	4.23
G23	-	-	-	-2.06	7.68	-	-	6.09	2.66	2.41	4.99	4.48	4.05	4.06
G24	11.02	8.61	6.17	-3.77	7.22	-	-	6.00	2.83	3.25	5.07	4.59	4.45	4.05
G25	11.29	8.73	6.04	-	8.19	-	-	6.22	2.64	2.41	4.75	4.26	4.19	4.26

Table S4. Proton chemical shifts for the structure of 5J05. Proton (20 °C) chemical shifts for sequence 5J05 in 20 mM NaPi (pH 6.8) aqueous buffer. Exchangeable-proton chemical shifts were obtained at 5° C while non-exchangeable proton chemical shifts were obtained at 20° C. H5'/H5'' protons are not stereo-specifically assigned

Residue	H1/H3	H21	H22	H8/H6	H2	CH_3	H1'	H2'	H2''	H3'	H4'	H5'	H5''
G1	12.161	11.039	6.408	7.358	-	-	5.761	2.824		4.871	4.156	-	-
G2	12.182	10.125	6.997	8.15	-	-	5.566	2.568	2.505	5.151	4.195	4.117	4.198
G3	11.414	10.363	6.594	7.827	-	-	6.311	2.789		5.708	4.451	4.289	4.17
T4	-	-	-	6.673	-	1.46	5.147	1.408	2.1	4.383	3.775	3.627	
T5	-	-	-	7.495	-	1.814	6.105	2.328		4.657	4.106	4.029	3.901
T6	-	-	-	6.675	-	0.694	5.802	1.658	1.949	4.486	3.869	3.693	
G7	10.905	9.879	7.186	8.091	-	-	5.78	3.267	2.596	4.575	4.142	-	-
G8	11.521	9.928	6.931	7.535	-	-	5.851	2.617		4.959	4.189	4.102	
G9	11.368	10.205	6.205	7.949	-	-	5.855	2.477	2.625	4.886	4.423	4.421	3.988
T10	-	-	-	6.881	-	1.69	5.362	1.177	2.005	4.401	3.814	3.341	3.344
T11	-	-	-	7.183	-	1.486	5.306	2.084	1.852	4.497	3.498	3.447	3.495
T12	-	-	-	7.076	-	1.424	5.404	1.565	2.218	4.649	4.398	2.657	4.264
T13	-	-	-	7.569	-	1.673	6.21	2.019	2.312	4.473	3.044	2.731	3.044
G14	11.939	9.491	7.514	7.438	-	-	6.09	3.419	2.895	4.849	4.254	4.06	4.04
G15	11.313	9.813	6.614	7.511	-	-	6.074	2.599	2.792	5.097	4.425	-	-
G16	11.152	9.976	6.414	7.866	-	-	6.246	2.954	2.59	5.026	4.244	3.777	3.629
A17	-	-	-	7.446	7.664	-	5.927	1.959	2.76	4.882	4.293	4.161	
G18	11.326	10.416	5.848	7.887	-	-	5.992	2.516	2.784	4.891	4.386	4.015	
G19	10.893	8.673	6.641	7.32	-	-	5.995	3.544	2.825	5.035	4.267	4.159	4.251
G20	11.628	9.292	6.999	8.263	-	-	6.283	2.703	2.418	4.252	4.165	-	-

Table S5. Proton chemical shifts for the structure of 5J4W. Proton (20 °C) chemical shifts for sequence 5J4W in 100 mM Na+ (pH 6.8) aqueous buffer. Exchangeable-proton chemical shifts were obtained at 5° C while non-exchangeable proton chemical shifts were obtained at 20° C. H5'/H5'' protons are not stereo-specifically assigned.

Residue	H1/H3	H21	H22	H8/H6	CH_3	H1'	H2'	H2''	H3'	H4'	H5'	H5''
G1	12.17	10.76	6.48	7.39	-	5.86	2.74	2.81	4.99	4.19	4.18	4.19
G2	12.11	-	-	8.14	-	6.01	2.51	2.80	5.09	4.23	-	-
T3	-	-	-	7.84	1.97	6.36	2.40	2.61	4.38	4.10	3.08	3.09
T4	11.00	-	-	7.34	1.65	5.80	1.89	2.24	4.62	4.06	3.52	3.10
T5	10.33	-	-	7.22	1.45	5.95	2.38	2.23	4.59	3.57	4.10	
G6	11.96	-	-	7.60	-	6.08	3.42	2.78	4.37	4.19	-	-
G7	11.66	-	-	8.06	-	5.94	2.65	2.76	5.00	4.38	4.09	4.08
T8	10.49	-	-	7.04	1.78	5.50	1.45	2.25	4.45	4.01	3.61	3.60
T9	10.12	-	-	7.28	1.57	5.48	2.08	1.94	4.56	3.62	3.54	3.61
T10	9.78	-	-	7.16	1.54	5.43	1.75	2.37	4.46	-	-	-
T11	-	-	-	7.61	1.74	6.30	2.13	2.40	4.53	3.15	2.80	2.94
G12	12.11	-	-	7.45	-	6.10	3.45	2.98	4.99	-	4.14	4.12
G13	11.82	-	-	8.05	-	6.19	2.89	2.48	5.12	4.40	4.33	4.32
T14	-	-	-	7.82	2.00	6.24	2.58	2.24	-	4.32	-	-
T15	10.91	-	-	7.30	1.26	6.15	2.65	2.09	4.93	-	-	-
G16	11. 36	9.67	6.98	7.34	-	6.05	3.53	2.96	4.97	4.28	-	-
G17	11.81	-	-	8.27	-	6.31	2.73	2.48	-	4.29	-	-

Table S6. Proton chemical shifts for the structure of 5J4P. Proton (20 °C) chemical shifts for sequence 5J4P in 100 mM Na+ (pH 6.8) aqueous buffer. Exchangeable-proton chemical shifts were obtained at 5° C while non-exchangeable proton chemical shifts were obtained at 20° C. H5'/H5'' protons are not stereo-specifically assigned.

Residue	H1/H3	H21	H22	H8/H6	CH_3	H1'	H2'	H2''	H3'	H4'	H5'	H5''
G1	11.94	10.71	6.29	7.33	-	5.85	2.88	2.79	4.99	4.36	-	-
G2	12.25	10.39	6.83	8.16	-	6.01	2.69	2.68	5.09	4.34	4.21	4.19
T3	-	-	-	7.96	2.00	6.45	2.63	2.51	4.42	4.10	4.10	4.11
T4	-	-	-	7.30	1.57	5.99	2.12	1.97	4.55	4.10	3.92	3.92
T5	10.85	-	-	7.19	1.60	5.95	2.38	2.38	4.78	3.79	3.57	2.95
G6	11.93	9.90	7.03	7.55	-	6.03	3.33	3.32	4.24	4.17	4.10	3.79
G7	11.72	-	-	8.10	-	5.95	2.76	2.66	4.99	4.47	-	-
T8	10.65	-	-	7.06	1.77	5.53	2.27	1.53	4.45	4.03	3.51	3.62
T9	10.20	-	-	7.26	1.58	5.49	1.93	2.02	4.54	4.54	3.63	3.52
T10	9.90	-	-	7.18	1.54	5.43	2.34	2.06	4.71	4.43	3.99	3.98
T11	10.79	-	-	7.57	1.72	6.25	2.40	2.11	4.55	-	3.19	2.91
G12	11.82	9.53	7.59	7.09	-	6.12	3.18	3.42	4.96	4.72	4.13	4.14
G13	9.64	6.76	11.79	7.68	-	6.18	2.55	2.62	5.18	4.44	-	-
T14	11.15	-	-	7.43	1.44	6.04	2.50	2.40	4.93	4.19	-	-
T15	11.29	-	-	7.50	1.65	6.07	2.52	2.18	4.82	4.28	3.97	3.96
T16	9.81	-	-	7.91	2.08	6.37	2.50	2.23	4.88	4.51	4.27	4.14
G17	11.52	9.24	7.06	7.25	-	5.99	3.58	3.59	4.97	4.44	4.32	4.31
G18	11.74	-	8.34	8.28	-	6.38	2.52	2.78	-	-	-	-

Table S7. Proton and phosphorous chemical shifts for the structure of 2M6V. Proton and phosphorous (20 °C) chemical shifts for sequence 2M6V in 20 mM NaPi (pH 6.8) aqueous buffer. Exchangeable-proton chemical shifts were obtained at 5° C while non-exchangeable proton chemical shifts were obtained at 20° C. H5'/H5'' protons are not stereo- specifically assigned.

Residue	H1/H3	H21	H22	P	H8/H6	CH_3	H1'	H2'	H2''	H3'	H4'	H5'	H5''
G1	12.02	10.86	6.381	-	7.369	-	5.811	2.728	2.56	4.963	4.357	4.027	3.877
G2	11.9	-	-	-4.450	7.663	-	5.989	2.566	2.248	5.002	4.401	4.141	
G3	-	-	-	-3.952	8.023	-	5.85	2.646	2.521	4.922	4.462	4.332	4.162
T4	-	-	-	-3.470	7.271	1.867	5.455	2.023	1.92	4.558	3.789	4.35	3.928
T5	6.649	-	-	-4.243	6.876	1.274	5.498	2.332	1.757	4.467	3.64	3.499	2.635
G6	12.25	9.785	6.271	-4.871	7.347	-	5.906	2.828	2.627	5.079	4.194	4.355	3.987
G7	11.01	9.791	6.811	-4.732	7.54	-	5.95	3.161	2.654	4.934	4.33	4.232	4.184
G8	11.44	9.614	-	-4.232	8.048	-	5.951	2.718	2.639	4.991	4.493	4.397	4.089
T9	-	-	-	-3.895	7.076	1.795	5.606	2.232	1.525	4.513	4.034	3.631	3.527
T10	-	-	-	-4.089	7.283	1.576	5.45	2.078	1.913	4.579	3.51	3.639	3.55
T11	7.09	-	-	-4.562	7.227	1.56	5.489	2.419	1.758	4.753	4.493	4.087	3.998
T12	-	-	-	-3.192	7.64	1.771	6.327	2.463	2.1	4.595	3.091	3.002	2.902
G13	12.07	9.72	7.375	-4.079	7.549	-	6.177	3.547	3.088	5.034	4.9	4.532	4.215
G14	11.9	9.297	6.87	-4.394	7.876	-	6.283	2.821	2.514	5.218	4.427	4.391	
G15	-	-	-	-5.657	8.018	-	6.284	3.102	2.517	5.093	4.433	4.262	
T16	9.834	-	-	-5.623	7.141-	1.118	6.042	2.711	2.086	4.99	4.45	4.404	4.148
G17	11.56	9.615	7.065	-4.915	7.408	-	5.952	3.451	2.856	4.998	4.45	4.164	4.099
G18	11.74	-	-	-4.383	7.912	-	6.041	2.256		4.977	4.331	4.129	
G19	10.17	-	-	-4.463	8.014	-	6.092	2.662	2.603	4.831	4.211	4.316	4.172

Structural statistics tables

Table S8. NMR restraints and structural statistics for the structures of 2MFT.

NOE-derived distance restraints	Non-exchangeable	Exchangeable
Total	571	122
Intranucleotide NOEs	396	19
Sequential (i, i +1)	154	29
Long-range (i, >i +2)	21	74
Torsion angle restraints	0	
Hydrogen bond restraints[a]	8	
Structural Statistics NOE violations >0.3 Å	0	
Pairwise heavy atom rmsd (Å) all residues	1.06	

Table S9. NMR restraints and structural statistics for the structures of 2M6W.

NOE-derived distance restraints	Non-Exchangeable	Exchangeable
Total	645	379
Intranucleotide NOEs	427	69
Sequential (i, i +1)	175	76
Long-range (i, >i +2)	43	234
Torsion angle restraints	195	
Hydrogen bond restraints[a]	64	
Structural Statistics NOE		
violations >0.3 Å	0	
Pairwise heavy atom rmsd		
(Å) all residues	0.664	
Pairwise heavy atom rmsd		
(Å) structured[a]	0.554	

[a]Residues 1-4, 7-13, 15-18 and 20-24

Table S10. NMR restraints and structural statistics for the structures of 5J6U.

NOE-derived distance restraints	Non-exchangeable	Exchangeable
Total	1083	113
Intranucleotide NOEs	621	32
Sequential (i, i +1)	295	28
Long-range (i, >i +2)	20	53
Torsion angle restraints	0	
Hydrogen bond restraints	65	
Structural Statistics NOE violations >0.3 Å	0	
Pairwise heavy atom rmsd (Å) all residues	0.682	

Table S11. NMR restraints and structural statistics for the structures of 5J05.

NOE-derived distance restraints	Non- Exchangeable	Exchangeable
Total	428	94
Intranucleotide NOEs	335	38
Sequential (i, i +1)	78	11
Long-range (i, >i +2)	15	45
Torsion angle restraints	0	
Hydrogen bond restraints[a]	48	
Structural Statistics NOE violations		
>0.3 Å	0	
Pairwise heavy atom rmsd (Å) all		
residues	0.994	
Pairwise heavy atom rmsd (Å)		
structured[a]	0.517	

[a]Residues 4-6 and 10-13.

Table S12. NMR restraints and structural statistics for the structures of 5J4W.

NOE-derived distance restraints	Non-exchangeable	Exchangeable
Total	425	75
Intranucleotide NOEs	286	31
Sequential (i, i +1)	112	19
Long-range (i, >i +2)	27	56
Torsion angle restraints	0	
Hydrogen bond restraints	40	
Structural Statistics NOE violations		
>0.3 Å	0	
Pairwise heavy atom rmsd (Å) all		
residues	0.644	

Table S13. NMR restraints and structural statistics for the structures of 5J4P.

NOE-derived distance restraints	Non-exchangeable	Exchangeable
Total	532	85
Intranucleotide NOEs	361	27
Sequential (i, i +1)	135	16
Long-range (i, >i +2)	36	42
Torsion angle restraints	0	
Hydrogen bond restraints[a]	32	
Structural Statistics NOE violations		
>0.3 Å	0	
Pairwise heavy atom rmsd (Å) all		
residues	0.410	

Table S14. NMR restraints and structural statistics for the structures of 2M6V.

NOE-derived distance restraints	Non-Exchangeable	Exchangeable
Total	347	195
Intranucleotide NOEs	226	31
Sequential (i, i +1)	100	44
Long-range (i, >i +2)	21	120
Torsion angle restraints	167	
Hydrogen bond restraints[a]	36	
Structural Statistics NOE violations		
>0.3 Å	0	
Pairwise heavy atom rmsd (Å) all		
residues	0.853	
Pairwise heavy atom rmsd (Å)		
structured[a]	0.517	

[a]Residues 1-2, 4-9, 11, 13-14, and
